# TBM preferred to AlphaFold 3 for functional models of insect odorant receptors

**DOI:** 10.1016/j.csbj.2025.08.028

**Published:** 2025-08-26

**Authors:** Vaanathi Chidambara Thanu, Amara Jabeen, Spyros E. Zographos, Phillip W. Taylor, Shoba Ranganathan

**Affiliations:** a Applied BioSciences, Macquarie University, University, Sydney, NSW 2109, Australia; b Institute of Chemical Biology, National Hellenic Research Foundation, Athens, Greece

**Keywords:** Transmembrane proteins, Insect odorant receptors, Template-based modeling, AlphaFold, Fatty acid ligands, Molecular dynamics, Molecular docking, Receptor-ligand interactions, Functional mutation

## Abstract

Insect odorant receptors (iORs) are seven-transmembrane-domain (7TM) ion channels crucial for insect survival, playing key roles in insect behaviour such as foraging, pollination, social interaction, and prey recognition. Unlike G-protein coupled receptors (GPCRs), iORs are “inverted” in the membrane and function independently of accessory G proteins. iORs form complexes with a highly conserved co-receptor (Orco), allowing calcium ions to enter the cell upon ligand binding and are promising targets for pest control strategies. However, due to their structural complexity, experimental determination of iOR structures remains a challenge. This scarcity of experimental data underscores the urgent need for reliable computational modeling approaches to understand iOR structure and function. With the rise of the deep learning (DL) approach AlphaFold, currently in its third version, we explored whether detailed template-based modeling (TBM), using the few available experimental insect OR structures, is still required, instead of a quick AI-generated AlphaFold 3 (AF3) model. For six OR sequences from three insect orders, we compared TBM models with standard AF3 models, and AF3 models with lipid molecules, creating an artificial membrane. Our evaluation of functional mutagenesis data supports TBM models for iORs rather than AF3 models, for ligand discovery for insect pest-specific control using structure-based approaches.

## Graphical Abstract

**Figure:**
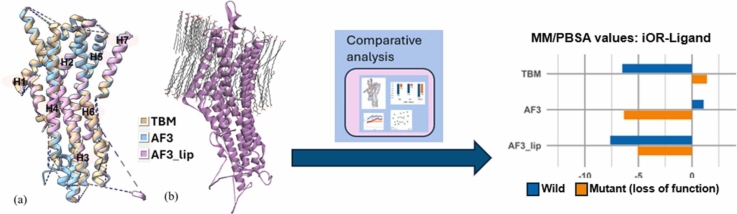

## 1 Introduction

Insect odorant receptors (iORs) are transmembrane proteins (TMPs) that play an important role in insect olfaction by detecting and responding to essential environmental cues, thereby supporting critical survival activities [1]. iORs are of particular interest in the context of pest control and insect behavior studies [2], [3], [4]. These receptors belong to the seven-transmembrane helix (7TM) protein family, resembling G protein-coupled receptors (GPCRs), but with an inverted topology, featuring an intracellular N-terminus and an extracellular C-terminus [5]. Unlike vertebrate ORs, iORs function as ligand-gated ion channels. Insects enhance their chemical sensitivity through heteromeric OR complexes that include a highly conserved odorant receptor co-receptor (Orco) [6], [7]. However, structural characterization of iORs remains limited due to the challenges associated with heterologous membrane protein expression and purification, in quantities suitable for experimental structure determination by cryo-EM studies. From 2019 to the present, only ten cryo-electron microscopy (cryo-EM) structures of insect olfactory receptors [8], [9], [10], [11] have been solved and deposited in the Protein Data Bank (PDB) [12]. (listed in Table S1).

Factors such as structural flexibility, low stability, low expression levels and partially hydrophobic surfaces make protein structure determination of membrane proteins particularly challenging, especially in terms of expression, purification, and crystallization [13]. With only ∼3.6 % [14] of the PDB representing TMPs, computational methods have emerged as promising alternatives to experimental approaches in advancing our understanding of these iORs. In 2020, during the CASP14 (Critical Assessment of Structure Prediction) competition [15], Google DeepMind and Isomorphic Labs Limited introduced AlphaFold2 (AF2) [16], a deep-learning-based model that significantly advanced protein structure prediction speed and accuracy without using templates. Following its success, the AlphaFold Protein Structure Database (AFDB) [17] has been created, with 214 million predicted models as of Jan 2024.

Artificial intelligence (AI)-based models can in many cases be very accurate; however, they generally do not consider the presence of ligands, point mutations, covalent modifications or environmental factors, and take protein–protein interactions and multiple conformations into account in a limited way [18], [19]. Also, AF2 accuracy decreases when dealing with novel or highly divergent sequences with limited homologous data. Furthermore, a recent study [20] indicates that it does not effectively capture structural changes caused by point mutations. Azzaz et al. [21] studied membrane protein structure prediction specifically and reported inconsistencies in AF2's prediction of transmembrane domain locations. Another study on aphid ORs [22] further showed when the AF2 models for these receptors were compared with TBM models, certain key residues in the ligand binding pocket were missing. While AlphaFold3 (AF3) [23], the latest version of AlphaFold, successfully introduced protein–protein complex prediction [24], it still faces notable limitations [20] and membrane proteins continue to pose difficulties, as their interactions with lipid bilayers complicate structural predictions [25]. To address this limitation, recently protein structure modeling including biomolecules such as lipids have been introduced to study the protein-lipid interactions and to improve accuracy [25], [26] making the AF3 server more applicable to real biological systems, along with a limited set of experimental ligands. However, the experimental structures used to train AF3 contain experimental PDB structures upto 30 September 2021 [23], possibly excluding the six 2024 iOR structures.

Insect olfaction in most species requires heteromeric assemblies of usually one Orco and a variable number of ORs, from only four in the damselfly to more than 350 in some ants [8]. Also, iOR sequences are very diverse, within and between species, with about 20 % amino-acid identity [8]. Although accurate modeling of targets from templates of less than 20 % sequence identity remains a challenge for TBM, this approach has been successfully used to predict iOR structures with accuracy [22], [27], [28], [29], [30], [31], [32], [33], [34], [35] as well as applied to ligand binding studies providing valuable insights into their structure and function. Although the iOR family is highly divergent and the available experimental structures are from evolutionarily distant organisms, these structures show a high level of structural similarity. The selection of templates based on various physical parameters [35], [36] and improved alignments [37] can allow for accurate modeling of structures with the limited templates. Based on this rationale, we recently developed iBio-GATS [34], a semi-automated workflow for improved template selection and alignment. Using iBio-GATS, we demonstrated that TBM was better than AF2 for predicting the structures of *D. melanogaster* odorant receptors, *Dm*OR59b using MhOR5 (PDB ID: 7LIG) as template and *Dm*Orco using *Ab*Orco (PDB ID: 6C70) as template, effectively capturing known mutagenesis data [31], [38]. While AF2 predicted the overall fold, the helix orientations in the predicted models were unable to capture the reported effect on ligand binding upon mutation of specific residues. Also, a recent study [39] on OR-ligand complexes in the fruit fly species, *Bactrocera dorsalis* and *Bactrocera minax* revealed that TBM models were more accurate than those from AF3 in capturing ligand affinities.

Given the large number of iORs in most insects of economic importance, we were keen to explore whether AF3 could be replace TBM for genome-scale iOR structural modeling. Building on insights from previous reports, the present study compares TBM and two versions of AF3 generated structural models: the standard version of AF3 (AF3 models) and AF3 with lipids (AF3_lip models) for selected iOR sequences. The aim of the study is to analyse how well these methods reproduce experimental mutagenesis data affecting ligand binding, and to gain a deeper understanding of their strengths and limitations. In addition to *Dm*Orco and *Dm*OR59b that were studied earlier [34], two more *D. melanogaster* iOR sequences, *Dm*OR85b and *Dm*OR22a with mutagenesis data [40], [41] were included. To further explore whether model performance is organism-dependent, we also included two additional insect species from different orders: *It*OR46 from a spruce bark beetle, *Ips typographus* (Coleoptera) [42] and *Of*OR3 from *Ostrinia furnacalis,* a moth (Asian corn borer, Lepidoptera) [43]. All organisms in this study were selected based on the availability of experimentally characterized mutational data (shown in Table 1), resulting mostly in loss of function, except for *Of*OR3 where gain of function has been characterized. Also, other than the Orco sequence, which is highly conserved in insects, all other iOR sequences showed 9–13 % sequence identity with their selected templates (Table S2). We directly compared the ligand binding capabilities of wild and mutant forms of the receptors, to understand the impact of specific mutations on receptor structure and function. The inclusion of species from different orders broadens the scope of our study and represents a step toward understanding iORs across a wider range of insect families.

**Table 1:** List of iOR sequences with their respective ligands, mutation studied, its functional effect on ligand binding and EC50 values or binding descriptors provided in the literature.

**No**	**Sequence**	**Ligand**	**Mutation**	**Effect on ligand binding**	**EC₅₀ (µM)**		**Reference**
					**Wild**	**Mutant**	
1	*Dm*Orco	VUAA1	F84A	Loss of function	95	105	[31]
2	*Dm*OR59b	DEET	V91A	Loss of function	Binding	Non-Binding	[38]
3	*Dm*OR85b	2-heptanone	F142C	Loss of function	70 ± 20	213 ± 61	[40]
4	*Dm*OR22a	methyl octanoate	M93I	Loss of function	Binding	Decreased binding	[41]
5	*It*OR46	(*S*)-(-)-ipsenol	T205A	Loss of function	1.98	Non-binding	[43]
6	*Of*OR3	(*E*)−11-tetradecenyl acetate	T148A	Gain of function	0.179 ± 0.080	0.0153 ± 0.0031	[42]

For each receptor, structural models were generated using TBM, AF3, and AF3_lip approaches. iBio-GATS selected the best template using all available iOR structures (Table S1) for building the TBM models. AF3 models were generated by the AF3 server. To approximate the lipid bilayer, 50 palmitic acid (PLM) and 50 oleic acid (OLA) molecules were added, being the maximum molecules allowed for each entity on the AF3 server. To study the experimental evidence, mutant versions of each receptor were also modeled. Thus, for each receptor, six models were generated, and they are denoted as

• TBM wild (*Wt*TBM) and TBM mutant (*Mt*TBM)
• AF3 wild (*Wt*AF3) and AF3 mutant (*Mt*AF3)
• AF3_lip wild (*Wt*AF3_lip) and AF3_lip mutant (*Mt*AF3_lip)

A total of 32 models across the four sequences of *D. melanogaster* ORs, OR46 from *I. typographus* and OR3 from *O. furnacalis* were generated, with four TBM models for wild and mutant *Dm*Orco and *Dm*OR59b from our earlier study [34]. The generated models were docked with their cognate ligands, and molecular dynamics (MD) simulations (3 replicates of 100 ns) were performed using explicit solvent and membrane conditions, following the use of 3 replicates as in a recent study [44] and adopting simulation times as in our previous study [34] Binding free energy (Δ*G*_*bind*_) given in kcal mol^−1^ was evaluated using MM/PBSA analysis, which is considered more accurate than MM/GBSA [45]. To explore the relative stability of the wild and mutant complexes, simulation trajectories were analyzed in terms of root-mean-square deviation (RMSD), root-mean-square fluctuation (RMSF), and ligand-binding interactions. By comparing the docking scores, energy values, structural stability and ligand interactions across all the models generated by the three approaches, we evaluated the strengths and limitations of TBM, AF3 and AF3-lip in predicting functional iOR models. The TBM models functionally characterized the mutational effects of ligand binding for the six iORs studied here. AF3 was able to predict the overall fold quite well, although the addition of lipid molecules did not improve the results significantly. Overall, the AF3 mutant models reported better binding energies than the wild type models for loss of function mutants and slightly lower values for the gain of function mutant, contrary to experimental findings [31], [38], [40], [41], [42], [43].

## 2 Materials and methods

**Fig. 1:**
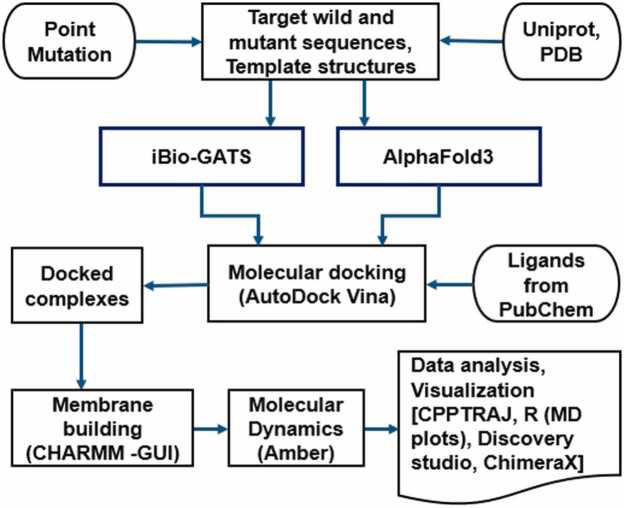
Workflow of the methodology used in this study.

Fig. 1 depicts the workflow used in this study, with descriptions provided in the subsections below.

### 2.1 Sequence retrieval

iOR sequences used in this study; *Dm*Orco, *Dm*OR59b, *Dm*OR85b, and *Dm*OR22a from *D. melanogaster, It*OR46 from *I. typographus* and *Of*OR3 from *O. furnacalis*, were retrieved in fasta format from the UniProt database [46]. Accession numbers and sequence details for the six iOR sequences are provided in Table S2. Templates for each iOR sequence were selected by iBio-GATS by searching all available iOR structures. The structures of selected templates, listed in Table 1, were subsequently downloaded in PDB format from the PDB database [12]. The sequences corresponding to the iOR template structures were extracted from their corresponding PDB files using Discovery studio (version 21.1.0.20298) [47] and saved in fasta format for generating the query-template alignments.

### 2.2 Ligands dataset

**Fig. 2:**
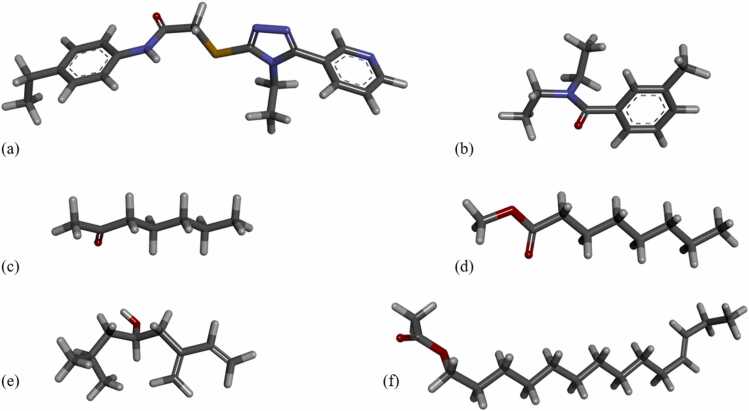
**The chemical structure of the ligands used in this study.** (a) VUAA1 (CID:1319135), (b) N, N-diethyl-meta-toluamide (DEET) (CID:4284), (c) 2-heptanone (CID:8051), and (d) methyl octanoate (CID:8091), (e) (*S*)-(−)-ipsenol (CID: 85712) and (f). (*E*)-11-tetradecenyl acetate (CID:5367650) in stick format and with atom colours (C: grey, H: white, N: blue, O: red and S: yellow).

The ligands used in this study: VUAA1/ORCORAM for *Dm*Orco, DEET for *Dm*OR59b, 2-heptanone for *Dm*OR85b, methyl octanoate for *Dm*OR22a, (*S*)-(−)-ipsenol for *It*OR46 and (*E*)-11-tetradecenyl acetate for *Of*OR3, were retrieved in SDF format from the Pubchem database [48]. The ligands (Fig. 2) were subsequently converted into PDB format using UCSF ChimeraX (v1.4) [49] for further use in this study.

### 2.3 Structure modeling

Structural models were generated using iBio-GATS [34] and the AF3 server [23]. Template-based modeling (TBM) was done using iBio-GATS, a semi-automated workflow designed for modeling iORs [34]. Briefly, iBio-GATS was downloaded from GitHub (https://github.com/CVaans/iBio-GATS), with pre-2024 iOR templates and additional templates downloaded for the six recent iOR structures. Each sequence was run through iBio-GATS for selecting the best match selection from the ten available structures (Table S1). The pairwise sequence alignments between each iOR sequence and its selected template were generated using AlignMe [50]. Based on iBio-GATS template selection and user preference, the best template was selected for modeling (listed in Table S2). Manual editing of the alignment generated by iBio-GATS was performed using MEGA [51] (Molecular Evolutionary Genetics Analysis; version 11) to remove gaps within helical segments. The software package, Modeller [52], integrated within iBio-GATS, generated five models for each iOR, and the model with the lowest objective function score was selected for further analysis. Point mutations were introduced for each iOR sequence based on experimental evidence and mutant models were generated afresh using iBio-GATS. The earlier models [34] obtained for *Dm*Orco and *Dm*OR59b wild type and mutant sequences were retained for comparison with AF3 models for ligand docking followed by MD simulation and analysis. All TBM models were checked for structural quality as detailed in our earlier study [34].

AF3 models were generated for each iOR sequence, using the AF3 web server [23]. The AF3 server currently provides a fixed set of 18 ligands with none of the iOR ligands in Fig. 2 nor the phospholipids constituting the biological membrane, to build receptor models with specific ligands present. It is also not possible to upload ligand structure files to the server in its present form. We therefore selected two available fatty acids, palmitic acid (PLM) and oleic acid (OLA) molecules from the AF3 server’s set of available ligands, to provide a lipid environment with saturated and unsaturated fatty acids. The membrane-embedded (AF3_lip) models incorporating PLM and OLA molecules were generated to check if the lipid environment made any difference to the standard AF3 models. AF3_lip models were generated on the AF3 server by including lipid molecules along with the input sequence. Specifically, 50 PLM and 50 OLA molecules were added from the list of available ligands, as this is the maximum allowed for each entity by the server. The addition of these lipid molecules was intended to represent the lipid bilayer environment of the native insect membrane composition. While it was possible to add additional sets of 50 PLM and OLA molecules, we limited the number of lipid molecules to 100, as larger numbers of lipids led to micelle-like structures, rather than increasing protein-lipid interactions ("Sparks of Chemical Intuition"-and Gross Limitations!-in AlphaFold 3 | Towards Data Science). The placement of the lipids was handled automatically by the AF3 server during model generation. The top-ranked models for each iOR sequence, as predicted by AF3, were selected and used for further analysis. Point mutations were introduced for each iOR sequence based on experimental evidence and mutant AF3 and AF3_lip models were generated using the web server, following the same protocol as for the wild-type sequences.

To assess the structural similarity between the different models generated through TBM, AF3, and AF3_lip, the three models obtained for each iOR sequence were structurally superimposed using the MatchMaker tool in UCSF ChimeraX (v1.4) [49], to optimize helical overlay.

### 2.4 Molecular docking studies

Molecular docking studies of all 36 models were conducted using AutoDock Vina 1.2.0 [54] for each iOR/Orco *Wt*TBM, *Mt*TBM, *Wt*AF3, *Mt*AF3, *Wt*AF3_lip and *Mt*AF3_lip models. Each receptor model was subjected to molecular docking with their respective ligands: VUAA1, DEET, 2-heptanone, methyl octanoate, (*S*)-(−)-ipsenol and (*E*)-11-tetradecenyl acetate (Fig. 2). Binding pockets were initially identified using FpocketWeb 1.0.1[55] followed by visual inspection to identify the pocket of interest. Receptor models and ligand structures were prepared by adding polar hydrogens, assigning Gasteiger charges and converted into PDBQT format using AutoDock Tools. Grid-based docking was performed, with the grid box centered on the predicted binding pocket. Multiple docking runs were performed, and the top-scoring pose for each complex was selected based on the lowest binding affinity (kcal/mol) (Table S3). Each top-ranked AutoDock result was converted into a protein–ligand complex PDB file using UCSF Chimera (v1.16) [53]. Protein-ligand binding interactions were visualized and analysed using BIOVIA Discovery Studio Visualizer [47] and UCSF ChimeraX [49].

### 2.5 Molecular dynamics (MD) simulations

MD simulations were performed to refine all predicted protein-ligand complexes within a membrane environment. Simulations were carried out using Amber 22 [56], with membrane systems constructed using CHARMM-GUI Membrane Builder [57] through the CHARMM-GUI server [58]. POPC and POPE represents the two major lipid classes in insect membranes and were therefore included in this study, in ratios as close to the membranes of the specific insects. Previous simulation studies [39], [59], [60] have included only these major lipids classes for membrane building, ignoring minor lipids as well as sterol molecules. For the *D. melanogaster* membrane, we followed the reported lipid composition [61], of 50 % 1-palmitoyl-2-oleoyl-sn-glycero-3-phosphoethanolamine (POPE) and 25 % 1-palmitoyl-2-oleoyl-sn-glycero-3-phosphatidylcholine (POPC), with the remaining 25 % being membrane-embedded proteins and carbohydrates. Accordingly, 42 POPE and 22 POPC molecules were added to each leaflet of the 64-lipid membrane, resulting in a 128-lipid bilayer with a POPE/POPC ratio of approximately 2:1. The *I. typographus* membrane [62] was best represented by 36 POPC and 28 POPE molecules per leaflet, to form a 128-lipid bilayer. For the *O. furnacalis* membrane [63], where phosphocholines (PCs) are the most abundant phospholipid class, 42 POPC and 22 POPE molecules were used per leaflet. The complexes were solvated with TIP3P water, and NaCl was added to a final ionic strength of 0.15 mM, with ion placement determined using the Monte Carlo method. Simulations were run under periodic boundary conditions that defined an average simulation box volume for each study is given in Table S4. Equilibration was performed under the NPT ensemble at constant pressure with a temperature set to 298.15 K, using the Nose–Hoover thermostat, approximating physiological conditions in insects. 100 ns simulations were conducted under the NVE ensemble, with no pressure control or barostat applied. The earlier models [34] generated for *Dm*Orco and *Dm*OR59b wild type and mutant sequences were retained and compared with MD results from AF3 and AF3_lip complexes. To increase the reliability of MD results, a minimum of three independent replicates of 100 ns were performed for each protein–ligand complex, based on the recent study of GPCRs [44], with additional replicates where necessary to achieve reasonably concordant results. The MM/PBSA binding free energy values from three most consistent replicates were averaged, and the corresponding standard deviations were calculated to assess the reliability of the results (Table S5).

### 2.6 MD analysis

Binding free energies (Δ*G*_*bind*_) given in kcal mol^−1^ were estimated using the Molecular Mechanics Poisson–Boltzmann Surface Area (MM/PBSA) method [64]. These calculations were performed using the CPPTRAJ module [65] in Amber 22. Trajectory analysis was carried out using CPPTRAJ and plotted using the MDplot [66] package in R [67] for visualization. The binding free energy values and the fluctuation data (measured as root mean square fluctuation; RMSF) for the residue at the mutation site were extracted, averaged over the replicates and plotted using the ggplot2 package [68] in R. RMSD values for the complex and the ligand over the last 10 ns were averaged over the MD replicates to compare the relative stability of the wild and mutant complexes.

## 3 Results

### 3.1 TBM captures the mutagenesis evidence

Structural models of *apo* iORs were generated with iBio-GATS and two variations of AF3 (without and with a lipid environment) using the AF3 server. For iBio-GATS, the final pairwise sequence alignment of each iOR sequence with its respective selected template for all the six sequences is shown in Figures S1-S6. While the templates for *Dm*Orco and *Dm*OR59b remained the same as reported earlier [34], the preferred template for *Dm*OR85b, *Dm*OR22a, and *It*OR46 was the pea aphid *Acyrthosiphon pisum* OR5 (PBD ID: 8Z9A) [10]. Also, the top selected template for *Of*OR3 was the jumping bristletail *Machilis hrabei* MhOR5 (PDB ID: 7LIG) [9]. All the models obtained were found to be of excellent quality, based on structure quality analysis, as in (data not shown).

**Fig. 3:**
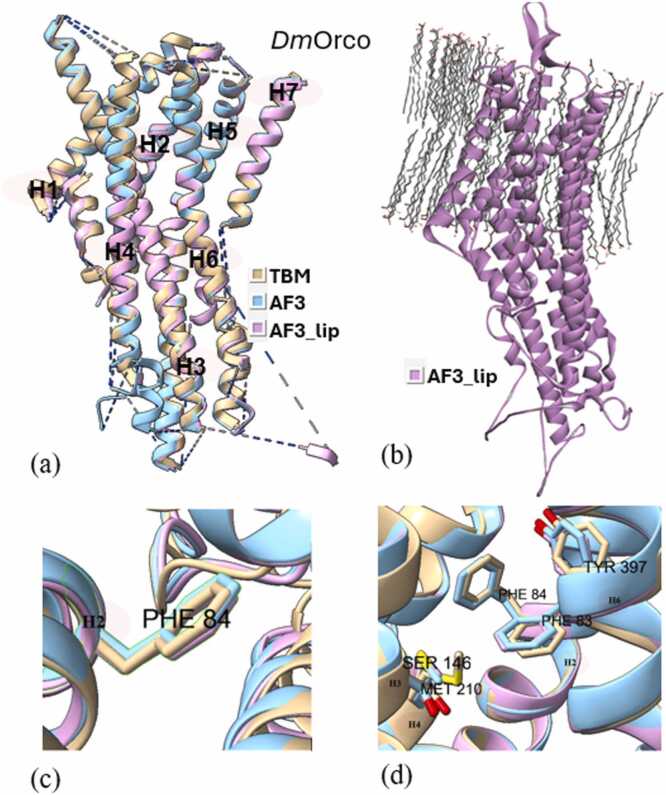
***Dm*****Orco models.** (a) Superimposition of the predicted models (TBM [34], AF3, AF3_lip), with helices numbered, (b) AF3 model with lipids (AF3_lip), (c) Detailed view of the conformation of the experimental mutagenesis evidence site (Phe 84) in the predicted models, and (d) Comparison of conserved binding pocket residues (Phe 83, Phe 84, Ser 146, Met 210, Tyr 400) [31] in the predicted models.

**Fig. 4:**
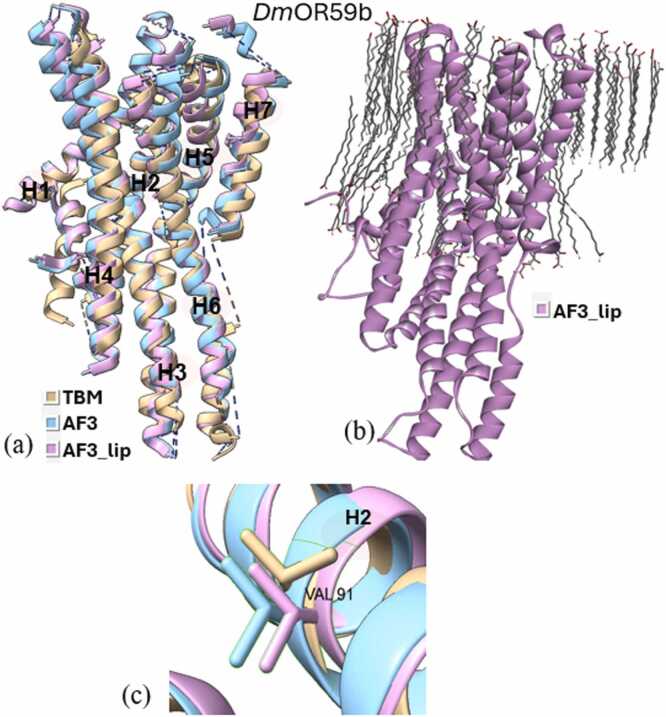
***Dm*****OR59b model**. (a) Superimposition of the predicted models (TBM [34], AF3, AF3_lip), with helices numbered, (b) AF3 model with lipids (AF3_lip) and (c) Detailed view of the conformation of the experimental mutagenesis evidence site (Val 91) in the predicted models.

**Fig. 5:**
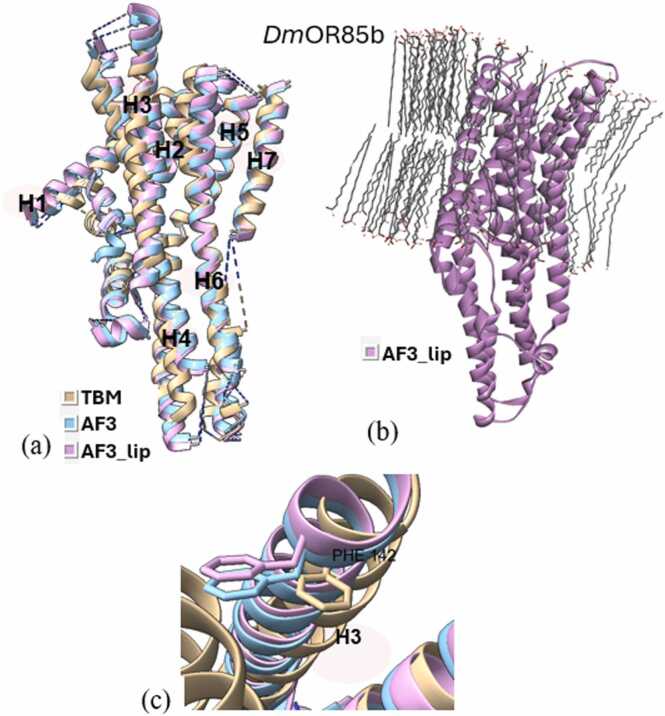
***Dm*****OR85b models.** (a) Superimposition of the predicted models (TBM, AF3, AF3_lip), with helices numbered, (b) AF3 model with lipids (AF3_lip) and (c) Detailed view of the conformation of the experimental mutagenesis evidence site (Phe 142) in the predicted models.

**Fig. 6:**
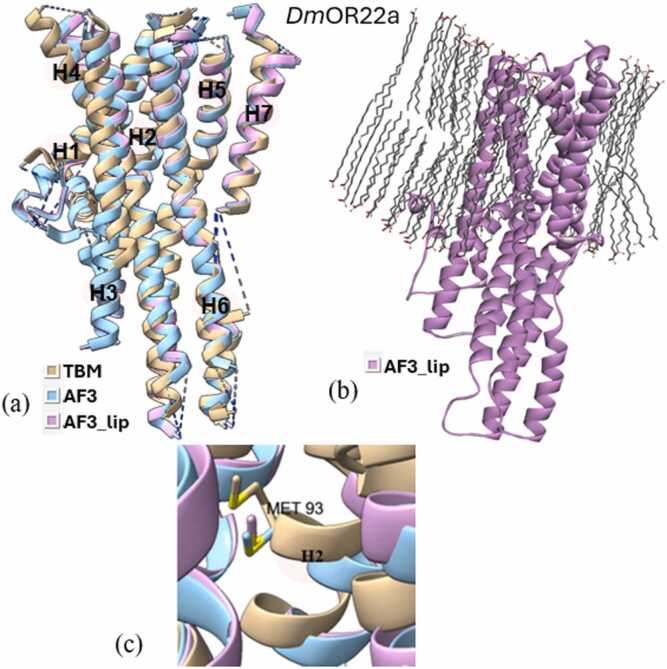
***Dm*****OR22a models.** (a) Superimposition of the predicted models (TBM, AF3, AF3_lip), with helices numbered, (b) AF3 model with lipids (AF3_lip) and (c) Detailed view of the conformation of the experimental mutagenesis evidence site (Met 93) in the predicted models.

**Fig. 7:**
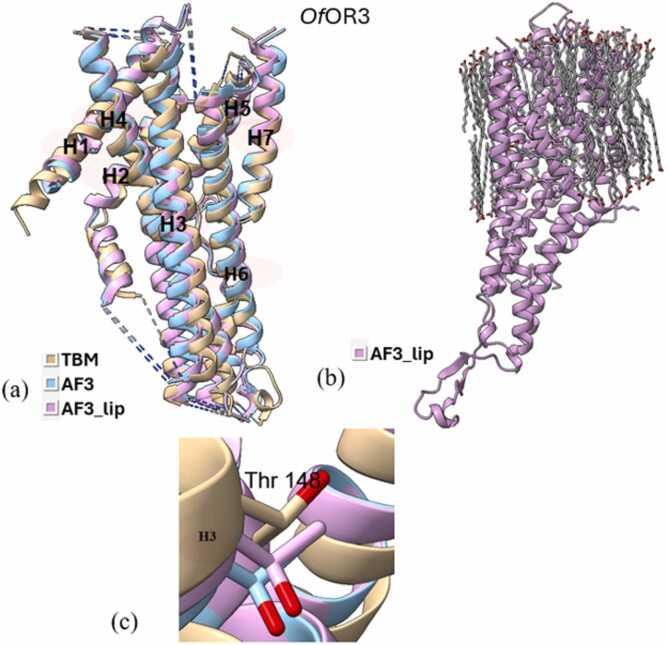
***It*****OR46 models.** (a) Superimposition of the predicted models (TBM, AF3, AF3_lip), with helices numbered, (b) AF3 model with lipids (AF3_lip) and (c) Detailed view of the conformation of the experimental mutagenesis evidence site (Thr 205) in the predicted models.

**Fig. 8:**
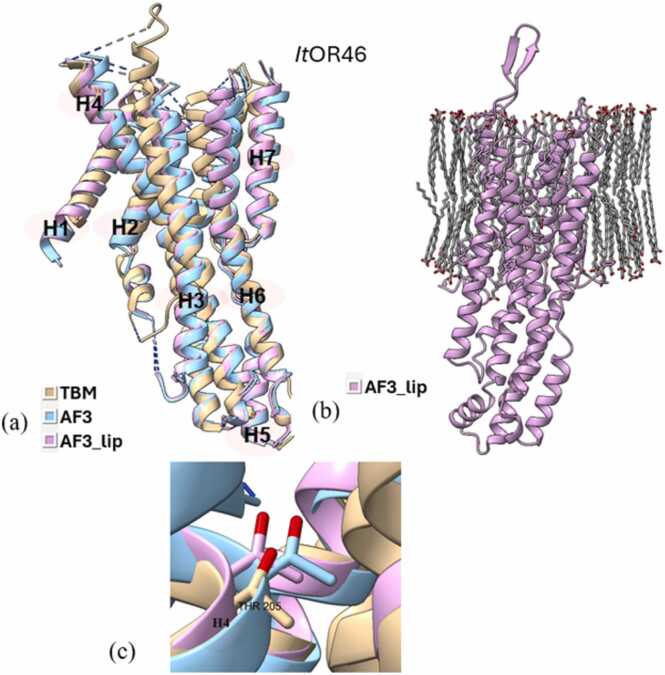
***Of*****OR3 models.** (a) Superimposition of the predicted models (TBM, AF3, AF3_lip), with helices numbered, (b) AF3 model with lipids (AF3_lip) and (c) Detailed view of the conformation of the experimental mutagenesis evidence site (Thr 148) in the predicted models.

Superimposition of all three types of models (TBM, AF3, and AF3_lip) for each wild type iOR sequence (Figs. 3a-8a) and their respective structural alignments (Figures S7-S12) revealed RMSD values for the helical regions within the expected range (<1.5 Å) of similar protein scaffolds. The AF3_lip models (Figs. 3b-8b) showed several lipid molecules clustering with each other in an uneven fashion, with only a few close to the hydrophobic TM helices, with 100 lipid molecules, comparable with earlier reports ("Sparks of Chemical Intuition"-and Gross Limitations!-in AlphaFold 3 | Towards Data Science).

### 3.2 Docking scores and post-docking interactions

Docking studies were done using all the predicted wild and mutant models of the OR sequences, with their respective ligands. The docking scores are presented in Table S4, and the post-docking interactions are given in Figures S13-S18. Docking of *Dm*Orco models with VUAA1 revealed that only the TBM complex captured the key residue 84 (Fig. 3c), with docking scores correlating with the experimental data. Neither AF3 nor AF3_lip complexes captured residue 84 in their binding pockets, and both approaches showed increased docking scores in the mutants, contradicting mutagenesis data. Also, only the TBM approach was able to capture all five conserved binding pocket residues (Phe 83, Phe 84, Ser146, Met210, and Tyr400)(Fig. 3d) reported by Pacalon et al. [31], which shows that TBM approach provided a more accurate prediction of the *Dm*Orco structure. Analysis of the post docking interactions of *Dm*OR59b with DEET, revealed that the *Wt*TBM complex captured the key residue Val91 (Fig. 4c), and its docking scores aligned with experimental evidence. In contrast, the *Wt*AF3 and *Mt*AF3 displayed docking scores with minimal reduction in binding affinity, despite capturing 91 in the binding site. Moreover, both the *Wt*AF3_lip and *Mt*AF3_lip complexes failed to capture residue91 in the binding site, and the *Mt*AF3_lip showed a stronger docking score than the *Wt*AF3_lip, further diverging from experimental results. In the case of *Dm*OR85b docked with 2-heptanone, all three approaches successfully identified 142 as a key residue (Fig. 5c). Both the *Wt*TBM and *Wt*AF3 complexes demonstrated stronger docking scores than their respective mutants (*Mt*TBM and *Mt*AF3), consistent with experimental trends. However, the *Mt*AF3_lip complex showed an increased binding affinity compared to *Wt*AF3_lip, contradicting to the experimental evidence. In *Dm*OR22a, all three modeling approaches successfully captured 93 as a key residue (Fig. 6c) when docked with methyl octanoate. Both the TBM and AF3 complexes were able to capture the difference of their wild and mutant in their docking scores, unlike the AF3_lip complex, which may be due to the influence of the lipid environment on the structural model.

*It*OR46 models docked with (*S*)-(−)-ipsenol successfully captured the key residue 205 (Fig. 7c). The TBM and AF3 approaches successfully captured the experimental evidence through their docking scores, reflecting loss of ligand binding affinity following mutation. However, the *It*OR46 AF3_lip complex did not reflect the same trend in the binding affinity, showing a slight gain of function following mutation. All three models of *Of*OR3 with the ligand (*E*)-11-tetradecenyl acetate captured 148 (Fig. 8c) as a key residue in the binding pocket. In the TBM models docked with (*E*)-11-tetradecenyl acetate, *Mt*TBM showed better binding compared to *Wt*TBM, supporting increased ligand affinity due to the mutation, which is consistent with the experimental gain of function result. The AF3 complex showed reasonable docking scores but showed a loss of function following mutation. The AF3_lip model showed better docking scores than TBM and AF3, with a slight increase in ligand affinity in *Mt*AF3_lip. To confirm the mutational effects indicated by docking scores, MD simulations were carried out on all complexes.

### 3.3 Energy values and MD analyses

**Fig. 9:**
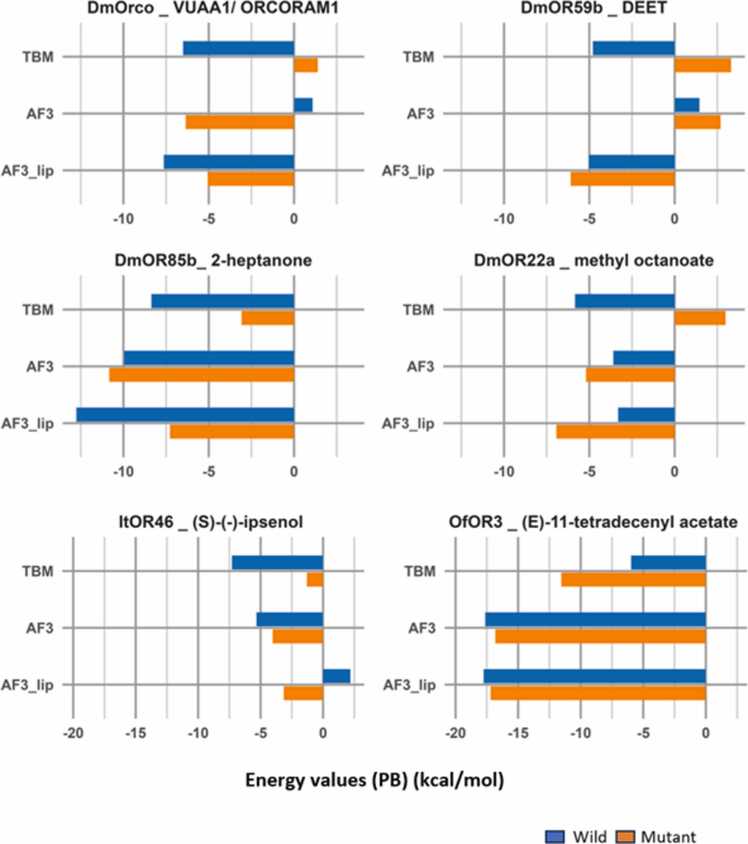
**Average binding affinity values**. MM/PBSA binding energy values (kcal mol^−1^) for wild and mutant TBM, AF3 and AF3_lip complexes for *Dm*Orco-VUAA1, *Dm*OR59b-DEET, *Dm*OR85b-2-heptanone, *Dm*OR22a-methyl octanoate, *It*OR46 with (S)-(-)-ipsenol and *Of*OR3 with (*E*)-11-tetradecenyl acetate.

The MM/PBSA binding energy values, averaged over the three replicates of local refinement of all the docked complexes are presented in Fig. 9, and their post-simulation interactions are in Figures S19-S24. The binding energy values for all replicates are available in Table S5.

Analysing the *Dm*Orco complexes with bound VUAA1, AF3 failed to capture the expected differences upon mutation. In contrast, the TBM complexes (Figure S19) reproduced the experimental loss of function due to the F84A mutation, similar to our earlier findings [34]. AF3_lip complexes showed strong binding of the ligand in wild and mutant receptors, although *Mt*AF3_lip showed a decrease binding affinity compared to *Wt*AF3_lip.

In *Dm*OR59b complexes with DEET (Figure S20), analysing the post-MD simulation interactions of AF3 and AF3_ lip complexes, revealed similar interactions to those observed in their pre-refinement state. Energy analysis showed that while both wild and mutant AF3 complexes were not energetically favourable to DEET binding, whereas both wild and mutant AF3_lip complexes bound the ligand well. TBM complexes alone exhibited a significant reduction in binding affinity after the incorporation of the V91A mutation, consistent with experimental findings and similar to our previous study [34].

Phe142 was identified as one of the key residues in the all the three complexes of *Dm*OR85b with 2-heptanone (Figure S21), and its interactions are lost in all mutant complexes similar to the pre-refinement interactions. Energy analysis of the TBM complexes revealed strong binding in *Wt*TBM complex which was seen to be reduced (less negative) in *Mt*TBM complexes. This trend was also reflected in the AF3_lip complexes, although wild and mutant complexes showed strong ligand binding. The AF3 complexes alone showed strong ligand binding despite F142C mutation.

In *Dm*OR22a complexes with methyl octanoate (Figure S22), all the complexes displayed similar interactions to their pre-refinement models. Energy analysis of the *Wt*TBM complexes had high binding affinity to methyl octanoate, which is considerably reduced in the *Mt*TBM complexes. However, both AF3 and AF3_lip complexes are favourable for methyl octanoate binding, although there is an increase in the binding affinity due to the M93I mutation.

Interaction analysis of wild and mutant *It*OR46 complexes with (*S*)-(−)-ipsenol (Figure S23) showed the presence of residue 205 with the TBM and AF3 models but not in the AF3_lip models. Energy analysis of the *Wt*TBM complex of *It*OR46 showed high ligand binding affinity, which was considerably reduced in the *Mt*TBM complex, consistent with experimental loss of function. A similar trend was observed with AF3 although both wild and mutant complexes were ligand binding, with the mutant showing slightly lower affinity for the ligand. In contrast, the *Wt*AF3_lip complex showed positive binding energy (repulsive) with (*S*)-(−)-ipsenol which become negative in the *Mt*AF3_lip complex.

The *Of*OR3 mutation T148A leads to a gain of function for the ligand, (*E*)-11-tetradecenyl acetate. The residue 148 was conserved in the binding site of all post-MD complexes, except the mutant TBM complex (Figure S24). Looking at the binding affinity, wild and mutant AF3 and AF3_lip complexes showed higher binding energy than the TBM complexes and no mutational effect was obvious. On the other hand, the TBM complexes captured the experimental evidence by showing a clear increase in binding affinity upon mutation.

Overall, all six TBM models showed binding affinity results consistent with experimental mutational data, for their cognate ligands. Complexes AF3 *It*OR46 with (*S*)-(−)-ipsenol, and AF3_lip *Dm*Orco with VUAA1 and *Dm*OR85b with 2-heptanone showed similar trends, with only modest decrease in ligand binding following mutation although wild and mutant complexes are both ligand binding. Complexes which show increased ligand binding on loss of function mutations include AF3 complexes of *Dm*OR85b with 2-heptanone and *Dm*OR22a with methyl octanoate and AF3_lip complexes of *Dm*OR59b with DEET and *Dm*OR22a with methyl octanoate. Both AF3 and AF3_lip *Of*OR3 complexes show marginal decrease in binding affinity for (*E*)-11-tetradecenyl acetate following the gain of function mutation. Models from this study that showed completely inverted ligand binding affinity results to experimental functional mutational evidence are the AF3 complex of *Dm*Orco with VUAA1 and the AF3_lip complex of *It*OR46 with (*S*)-(−)-ipsenol, while both wild and mutant AF3 complexes of DmOR59b with DEET are repulsive.

The RMSD values of trajectories over the last 100 ns of all docked complexes were averaged over the three replicates, and are presented in Figure S25, with ligand RMSD values in Figure S26. The TBM complexes showed higher RMSD values compared to the AF3 and AF3_lip complexes. The ligand RMSD values appear to be similar in all models.

**Fig. 10:**
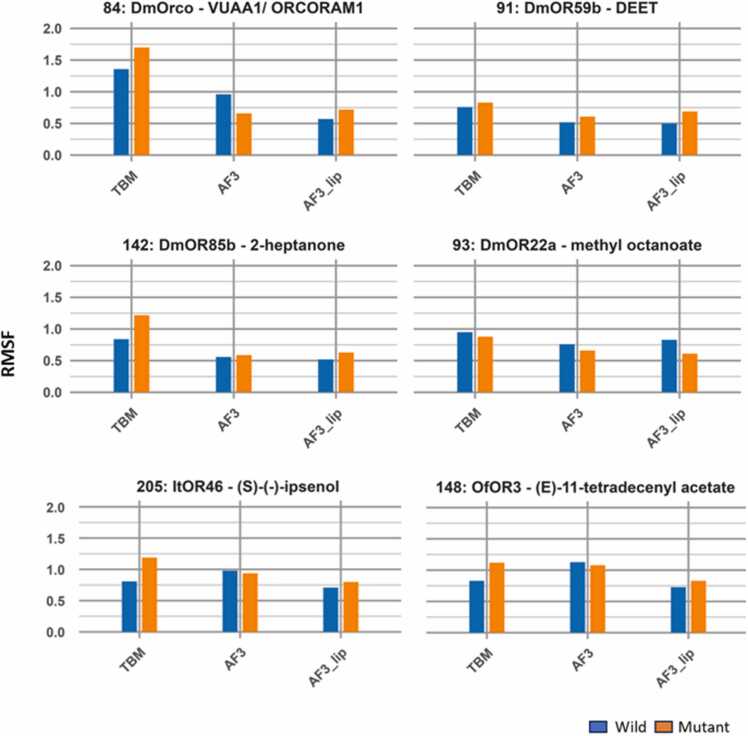
The RMSF values of the mutated residues in all 36 predicted complexes over the 100 ns MD simulations, averaged over three replicates.

The dynamic fluctuations of the residue that is mutated in each iOR, as measured by the RMSF values averaged over the three replicates, is shown in Fig. 10. Replacement of Phe or Thr by smaller amino acids, Ala or Cys resulted in the mutants showing larger RMSF values in the TBM complexes of *Dm*Orco with VUAA1, *Dm*OR85b with 2-heptanone, *It*OR46 with (*S*)-(−)-ipsenol and *Of*OR3 with (*E*)-11-tetradecenyl acetate, while AF3 and AF3-lip complexes showed little change or less fluctuation of the mutated residue. For the V91A mutation of *Dm*OR59b with DEET and the M93I mutation of *Dm*OR22a with methyl octanoate, there is limited change in the residue RMSF values for all models.

## 4 Discussion

Advances in AF modeling have significantly improved protein structure prediction, particularly for soluble proteins where AF has demonstrated remarkable accuracy often comparable to those obtained through experimental methods [69]. AF uses deep learning to predict protein structure directly from sequence. It constructs a multiple sequence alignment (MSA), extracts the information, and applies neural network architecture to build a protein structural model based on the extracted information. However, membrane proteins still present significant challenges in structural biology compared with soluble proteins. The lipid-rich environment [70], scarcity of training data for transmembrane proteins [71], and the dynamic flexibility of membrane-embedded regions [72] challenge the accuracy of such predictions. For membrane proteins such as (iORs), obtaining accurate 3D models is crucial to identify selective ligands, as structural insights are critical for obtaining a deep understanding of the binding pocket architecture and the mechanism of ligand recognition.

As transmembrane (TM) regions of proteins are subject to strong evolutionary constraints, leading to slower divergence rates compared to their extramembrane regions and soluble proteins. Despite this, TM regions often exhibit low sequence conservation, making MSAs challenging. Since AF relies heavily on MSAs for accurate structure prediction, this can lead to reduced prediction accuracy in the TM region [73]. Additionally, AF’s limitations [20] in understanding the specific context of membrane environments can result in misplaced helices, distorted loops, or incorrect topologies. Therefore, its predictions for membrane proteins such as iORs often require further refinement or validation through complementary methods. On the other hand, TBM remains a valuable approach for modeling membrane proteins, when structurally similar templates are available. A 2013 study on the relation between sequence and structure [74] demonstrated that TM regions can maintain structural conservation, even at sequence identities as low as 10 %, a threshold considered unreliable for globular proteins. This observation underscores the importance of structural information in aligning transmembrane segments correctly, prior to model building. Consequently, structure-based alignment methods become essential for accurate predictions, particularly for poorly characterized families such as iORs, with limited structural data. When structurally similar templates guide the modeling of unknown but functionally similar sequences, TBM can offer reliability, particularly when the query protein shares low sequence identity with available templates. This study aimed to evaluate the reliability and structural accuracy of two versions of AF3 and TBM approaches in predicting iOR models by focussing on the mutation data supported by experimental evidence. Given that AF3 and TBM employ distinct approaches, their structural predictions can differ significantly. By comparing models generated by these tools, particularly in terms of topology, transmembrane orientation, and binding pocket architecture, and analysing protein-ligand interaction based on mutational data with experimental evidence, we have provided an evaluation of their performance in predicting iOR structural models.

In this study, we selected six ORs with available mutagenesis data, from *D. melanogaster*, a beetle ORs. For TBM-based modeling, we utilized the iBio-GATS workflow, which is based on biophysical parameters and specifically designed for insect ORs. For AF3 predictions, we employed the AF3 server, and separately incorporated lipid molecules during modeling to approximate the membrane environment. We compared the quality of the three resulting models: TBM and two versions of AF3: AF3, and AF3_lip, by analysing their predictive reliability based on mutagenesis data with experimental evidence. For analysing experimental evidence, we performed molecular docking of each model with its respective ligand, followed by (MD simulations to evaluate the stability of the protein-ligand complexes. Also, we have carried out 3 ×100 ns replicates to generate at least three concordant MM/PBSA values, given the size of the system. Key parameters such as the docking score, binding free energy, RMSD and RMSF were calculated for the protein-ligand complexes.

Five of the six iOR sequences presented in this study show <15 % sequence identity to any available template structure. The TBM models generated using iBio-GATS performed better in terms of structural accuracy than the AF3 and AF3_lip models, even in the case *Dm*Orco, which has 55 % sequence identity to its template structure. The TBM models effectively captured key structural features of all six iORs, including the TM regions, ligand binding sites, and the effects of mutation. Also, TBM models demonstrated stable protein-ligand interactions during MD simulations, correlating with the experimental evidence. On the other hand, although AF3 and AF3_lip show good structural quality, they lack functional relevance. AF3 models did not consistently align with experimental data, especially in capturing the effects of mutation which is shown by docking scores and free binding energy values. Although AF3 and AF3_lip models did not show large fluctuations consistent with similar reports [44], [73], the effect of mutation was not reflected in their structural stability. While AF3_lip models showed some improvements in terms of stability due to the lipid environment, they still struggled to address the inaccuracies observed in AF3 predictions. Also, the integration of lipid molecules in AF3 predictions, does not fully compensate for the challenges in predicting accurate topology and ligand binding, particularly when mutations are involved. A recent study suggested that AF3 predicts functionally high-quality models for some insect odorant receptors (iORs) [75], while for the iORs studied here, TBM provides accurate models. The results of this study suggest the importance of structure-based methods, such as TBM, to accurately model membrane proteins like iORs, where experimental data is limited. Furthermore, as AF3 did not incorporate all the latest iOR templates, which were solved after its release, future versions of AF will definitely have improved accuracy for membrane protein structure prediction. Further refinement in deep learning models will definitely result in accurate predictions for membrane proteins, by incorporating ligands such as POPC and POPE to better recreate an *in silico* membrane environment.

In conclusion, AF has been called a game changer achieving accuracies comparable to experimental structures, especially for rigid, well-folded globular proteins [76]. However, for other classes of proteins, AF predicted models should be approached with a reasonable degree of caution and regarded as starting points for testable hypotheses rather than “as *de facto* ground truth structures” [76]. These hypothetical models should be guided by, and ideally validated against, experimental data such as mutational functional studies and *in vitro* or *ex vivo* ligand-binding assays, particularly in the context of structure-based ligand discovery. To this end, the iBio-GATS models generated in this work demonstrate better performance compared to AF3 models for the insect ORs studied here capturing five instances of loss of function and one instance of gain of function. TBM can therefore be employed for the discovery of novel control agents targeting insect species of both agricultural and public health importance. As an example, similar studies on *Anopheles gambiae* Orco–ligand complexes, guided by existing ligand-binding and functional data [4], are currently underway.

## CRediT authorship contribution statement

**Amara Jabeen:** Writing – review & editing, Visualization, Supervision, Resources. **Vaanathi Chidambara Thanu:** Writing – original draft, Visualization, Software, Methodology, Investigation, Formal analysis, Conceptualization. **Shoba Ranganathan:** Writing – review & editing, Visualization, Supervision, Resources, Project administration, Conceptualization. **Spyros E. Zographos:** Writing – review & editing, Validation. **Phillip W. Taylor:** Writing – review & editing, Supervision.

## Declaration of Competing Interest

The authors declare that they have no known competing financial interests or personal relationships that could have appeared to influence the work reported in this paper.

## Footnotes

Appendix A Supplementary data associated with this article can be found in the online version at doi:10.1016/j.csbj.2025.08.028.
