# Supplementary material for: TBM preferred to AlphaFold 3 for functional models of insect odorant receptors
Source: Comput Struct Biotechnol J. 2025 Aug 26;27:3908–18. doi: 10.1016/j.csbj.2025.08.028 (PMC12799948; doi:10.1016/j.csbj.2025.08.028)
Supplement: Supplementary file 1 — Supplementary material [file mmc1.pdf]

Supporting Information

List of Figures:

Figure S1. Final alignment of *Dm*Orco with *Ab*Orco (PDB ID:6C70)

Figure S2. Final alignment of *Dm*OR59b with *Mh*OR5 (PDB ID:7LIG)

Figure S3. Final alignment of *Dm*OR85b with *Ap*OR5 (PDB ID:8Z9A)

Figure S4. Final alignment of *Dm*OR22a with *Ap*OR5 (PDB ID:8Z9A)

Figure S5. Final alignment of *It*OR46 with *Ap*OR5 (PDB ID:8Z9A)

Figure S6. Final alignment of *Of*OR3 with *Mh*OR5 (PDB ID:7LIG)

Figure S7. Sequence alignment of the predicted *Dm*Orco models derived from structural superposition. The mutagenesis evidence site (Phe 84) is marked in black.

Figure S8. Sequence alignment of the predicted *Dm*OR59b models derived from structural superposition. The mutagenesis evidence site (Val 91) is marked in black.

Figure S9. Sequence alignment of the predicted *Dm*OR85b models derived from structural superposition. The mutagenesis evidence site (Phe 142) is marked in black.

Figure S10. Sequence alignment of the predicted *Dm*OR22a models derived from structural superposition. The mutagenesis evidence site (Met 93) is marked in black.

Figure S11. Sequence alignment of the predicted *It*OR46 models derived from structural superposition. The mutagenesis evidence site (Thr 205) is marked in black.

Figure S12. Sequence alignment of the predicted *Of*OR3 models derived from structural superposition. The mutagenesis evidence site (Thr 148) is marked in black.

Figure S13. 2D view of the binding pocket interactions for predicted models of *Dm*Orco with VUAA1.

Figure S14. 2D view of the binding pocket for interactions predicted models of *Dm*OR59b with DEET.

Figure S15.2D view of the binding pocket interactions for predicted models of *Dm*OR85b with 2-heptanone.

Figure S16. 2D view of the binding pocket interactions for predicted models of *Dm*OR22a with methyl octanoate.

Figure S17. 2D view of the binding pocket interactions for predicted models of *It*OR46 with (S)-(−)-ipsenol.

Figure S18. 2D view of the binding pocket interactions for predicted models of *Of*OR3 with (E)-11-tetradecenyl acetate.

Figure S19. Interactions of VUAA1 after local refinement with all predicted models of *Dm*Orco.

Figure S20. Interactions of DEET after local refinement with all predicted models of *Dm*OR59b.

Figure S21. Interactions of 2-heptanone after local refinement with all predicted models of *Dm*OR85b.

Figure S22. Interactions of methyl octanoate after local refinement with all predicted models of *Dm*OR22a.

Figure S23. Interactions of (S)-(−)-ipsenol after local refinement with all predicted models of *It*OR46.

Figure S24. Interactions of (E)-11-tetradecenyl acetate after local refinement with all predicted models of *Of*OR3.

Figure S25. Receptor RMSD values of all 36 complexes over 100 ns trajectories

Figure S26. Ligand RMSD values of all 36 complexes over 100 ns trajectories.

<u>List of Tables:</u>

Table S1: Experimental structures available for insect odorant receptors (iORs).

Table S2. List of iOR sequences and their respective selected templates used in this study.

Table S3. Docking scores of all 36 predicted models in this study.

Table S4. System size of all 36 predicted complexes in this study.

Table S5. MM/PBSA values (PB) and Standard Deviation (SD) of last 10 ns of the complexes (CSD) and their respective ligands (LSD) in all 36 complexes in this study.

```
                          10        20        30        40 He1    50        60
6C70_AbOrco      -------FKHQGLVADLLPNIRVMQGVGHFMFNYYSEGKKFPHRIYCIVTLLLLLLQYGM
Q9VNB5_DmOrco    MTTSMQPSKYTGLVADLMPNIRAMKYSGLFMHNF-TGGSAFMKKVYSSVHLVFLLMQFTF
                          70        80        90 He2    100       110       120
6C70_AbOrco      MAVNLMMESDDVDDLTANTITMLFFLHPIVKMIYFPVRSKIFYKTLAIWNNPNSHPLFAE
Q9VNB5_DmOrco    ILVNMALNAEEVNELSGNTITTLFFTHCITKFIYLAVNQKNFYRTLNIWNQVNTHPLFAE
                          130 He3   140       150       160       170       180
6C70_AbOrco      SNARFHALAITKMRRLLFCVAGATIFSVISWTGITFIEDS---------------PIPRL
Q9VNB5_DmOrco    SDARYHSIALAKMRKLFFLVMLTTVASATAWTTITFFGDSVKMVVDHETNSSIPVEIPRL
                          190       200 He4   210       220       230       240
6C70_AbOrco      MIRTFYPFNAMSGAGHVFALIYQFYYLVISMAVSNSLDVLFCSWLLFACEQLQHLKAIMK
Q9VNB5_DmOrco    PIKSFYPWNASHGMFYMISFAFQIYYVLFSMIHSNLCDVMFCSWLIFACEQLQHLKGIMK
                          250       260       270       280       290       300
6C70_AbOrco      PLMELSAT----------------------------------------------------
Q9VNB5_DmOrco    PLMELSASLDTYRPNSAALFRSLSANSKSELIHNEEKDPGTDMDMSGIYSSKADWGAQFR
                          310       320       330       340 He5   350       360
6C70_AbOrco      -------------------------GLTKKQEMLVRSAIKYWVERHKHVVRLVTAVGDAY
Q9VNB5_DmOrco    APSTLQSFGGNGGGGNGLVNGANPNGLTKKQEMMVRSAIKYWVERHKHVVRLVAAIGDTY
                          370       380       390 He6   400       410       420
6C70_AbOrco      GVALLLHMLTTTITLTLLAYQATKVNGVNVYAATVIGYLLYTLGQVFLFCIFGNRLIEES
Q9VNB5_DmOrco    GAALLLHMLTSTIKLTLLAYQATKINGVNVYAFTVVGYLGYALAQVFHFCIFGNRLIEES
                          430       440       450 He7   460       470       480
6C70_AbOrco      SSVMEAAYSCHWYDGSEEAKTFVQIVCQQCQKAMSISGAKFFTVSLDLFASVLGAVVTYF
Q9VNB5_DmOrco    SSVMEAAYSCHWYDGSEEAKTFVQIVCQQCQKAMSISGAKFFTVSLDLFASVLGAVVTYF

6C70_AbOrco      MVLVQLK
Q9VNB5_DmOrco    MVLVQLK
```

Figure S1. Final alignment of *Dm*Orco with *Ab*Orco (PDB ID:6C70)

Pairwise alignment of *Dm*Orco with *Ab*Orco (PDB ID:6C70) obtained from AlignMe, reproduced from [33] . Helices 1 to 7 are marked in red for the template and on green for the target sequence.

```
                           10        20        30        40  He1   50        60
7LIG_MhOR5        ------------DDYIHLRKWIKRIGIILRISGHWPFRLPHEKRNQHKSKFRQVYSCLVI
Q9W1P8_DmOR59b    MAVFKLIKPAPLTEKVQSRQGNIYLYRAMWLIGWIP---PKEGVLRYVYLFWTCVPFAFG
                           70        80        90       100  He2  110       120
7LIG_MhOR5        TLGFITCSCYCIGLCLS----ESIAQALNNITVTSYFLQSCVCYVSFIINSRKLETLFNY
Q9W1P8_DmOR59b    VFYLPVGFIISYVQEFKNFTPGEFLTSLQVCINVYGASVKSTITYLFLWRLRKTEILLDS
                          130       140       150       160  He3  170       180
7LIG_MhOR5        LFENEVVGCPRGYKMSSIKTTLFRCKFVAFSLGILSFFGWLMWTLLPLAVLVVDQTSLRF
Q9W1P8_DmOR59b    LDK-------RLANDSDRERIHNMVARCNYAFLIYSFIYCGYAGSTFLSYALSGRPPWSV
                          190       200       210 He4  220        230       240
7LIG_MhOR5        VEAWYPFDTTTSPMNEVIAIYEAVAMIFLITAPMSSDIMFCVLMIFIVEHLKCLGMAIEC
Q9W1P8_DmOR59b    YNPFIDWR-DGMGSLWIQAIFEYITMSFAVLQDQLSDTYPLMFTIMFRAHMEVLKDHVRS
                          250       260       270 He5  280        290       300
7LIG_MhOR5        TLKGD-------ATSLCNIVDSHVKIYRTMEIVQSVYSSYFATLFFTSCLAVCALAYFLA
Q9W1P8_DmOR59b    LRMDPERSEADNYQDLVNCVLDHKTILKCCDMIRPMISRTIFVQFALIGSVLGLTLVNVF
                          310       320 He6  330       340        350       360
7LIG_MhOR5        ATSTSFTRVPGMVLYLMYIFLRIFLLCLLATEVAEQGLNLCHAGYSSKLVLASDHVRSTI
Q9W1P8_DmOR59b    FFS-NFWKGVASLLFVITILLQTFPFCYTCNMLIDDAQDLSNEIFQSNWVDAEPRYKATL

                          370 He7  380       390       400        410
7LIG_MhOR5        QAIATRAQIPLSITGARFFTVNLSFLASMAGVMLTYFIVLLQVN------
Q9W1P8_DmOR59b    VLFMHHVQQPIIFIAGGIFPISMNSNITVAKFAFSIITIVRQMNLAEQFQ
```

Figure S2. Final alignment of *Dm*OR59b with *Mh*OR5 (PDB ID:7LIG)

Pairwise alignment of *Dm*OR59b with *Mh*OR5 (PDB ID:7LIG) obtained from AlignMe , reproduced from [33]. Helices 1 to 7 are marked in red for the template and in green for the target sequence.

```
                          10        20        30        40   He1  50        60
8Z9A_ApOR5         ----------------IDTINMFLQMTGCTDSKAMLYLTYFEFLITFYYLIATYASIVHF
Q7VHQ7_DmOR85b     MEKLMKYASFFYTAVGIRP----YTNGEESKMNKLIFHIVFWSNVINLSFVGLFESIYVY
                          70        80 He2     90       100       110       120
8Z9A_ApOR5         EQS----VTIQLFALLCMLIECVILLNITFRLYHKNHIREMHQYSR--------------
Q7VHQ7_DmOR85b     SAFMDNKFLEAVTALSYIGFVTVGMSKMFFIRWKKTAITELINELKEIYPNGLIREERYN
                         130 He3   140       150       160       170       180
8Z9A_ApOR5         RLGIPDSYRSVINVITKYHLIASNIFVVFPVTYAIFCDSVRVGD--PFTFPFLDVLPMHT
Q7VHQ7_DmOR85b     LPMYLGTCSRISLIYSLLYSVLIWTFNLFCVMEYWVYDKWLNIRVVGKQLPYLMYIPWKW
                         190       200       210 He4   220       230       240
8Z9A_ApOR5         DNLAIYACKYLVYAISVYIAHVELCFINTTFIYYVGVLKHRLETIVQTIGEAFADN----
Q7VHQ7_DmOR85b     QDNWSYYPLLFSQNFAGYTSAAGQISTDVLLCAVATQLVMHFDFLSNSMERHELSGDWKK
                         250       260 He5   270       280       290       300
8Z9A_ApOR5         DEQKFKYAIIQHQKLLSYFNTMKIVFSKPILLSMSFNAIYFGLTTSFVIQAIRGYINQAI
Q7VHQ7_DmOR85b     DSRFLVDIVRYHERILRLSDAVNDIFGIPLLLNFMVSSFVICFVGFQMTV----GVPPDI
                         310       320 He6   330       340       350       360
8Z9A_ApOR5         LSICIASSAAAVINITIYTFYGSELMDLHDKILHVLFDNAFFYVSKSFKSSILIMMTRVT
Q7VHQ7_DmOR85b     VVKLFLFLVSSMSQVYLICHYGQLVADASYGFSVATYNQKWYKADVRYKRALVIIIARSQ
                         370       380 He7   390
8Z9A_ApOR5         IPLKFTVGYIFTINLNLLLKILKMSYTVLNVLLSSET-
Q7VHQ7_DmOR85b     KVTFLKATIFLDITRSTMTDLLQISYKFFALLRTMYTQ
```

Figure S3. Final alignment of *Dm*OR85b with *Ap*OR5 (PDB ID:8Z9A)

Pairwise alignment of *Dm*OR85b with *Ap*OR5 (PDB ID:8Z9A) obtained from AlignMe. Helices 1 to 7 are marked in red for the template and in green for the target sequence.

```
                      10        20        30        40        50   He1  60
8Z9A_ApOR5      ------------------------------------IDTINMFLQMTGCTDSKAMLYLTY
P81909_DmOR22a  MLSKFFPHIKEKPLSERVKSRDAFIYLDRVMWSFGWTEP------ENKRWILPYKLWLAF
                      70        80        90        100       110  He2  120
8Z9A_ApOR5      FEFLITFYYLIATYASIVHFEQS--VTIQLFALLCMLIECVILLNITFRLYHKNHIREMH
P81909_DmOR22a  VNIVMLILLPISISIEYLHRFKTFSAGEFLSSLEIGVNMYGSSFKCAFTLIGFKKRQEAK
                      130       140       150  He3 160       170       180
8Z9A_ApOR5      QYSR---RLGIPDSYRSVINVITKYHLIASNIFVVFPVTYAIFCDSVRVGDPFTFPFLDV
P81909_DmOR22a  VLLDQLDKRCLSDKERSTVHRYVAMGNFFDILYHIFYSTFVVMNFPYFLLERR-HAWRMY
                      190       200  He4  210       220       230       240
8Z9A_ApOR5      LPMHTDNLAIYACKYLVYAISVYIAHVELCFINTTFIYYVGVLKHRLETIVQTIGEAFAD
P81909_DmOR22a  FPYIDSDE-QFYISSIAECFLMTEAIYMDLCTDVCPLISMLMARCHISLLKQRLRNLRSK
                      250       260  He5  270       280       290       300
8Z9A_ApOR5      N------DEQKFKYAIIQHQKLLSYFNTMKIVFSKPILLSMSFNAIYFGLTTSFVIQAIR
P81909_DmOR22a  PGRTEDEYLEELTECIRDHRLLLDYVDALRPVFSGTIFVQFLLIGTVLGLSMINLMF---
                      310       320       330  He6  340       350       360
8Z9A_ApOR5      GYINQAILSICIASSAAAVINITIYTFYGSELMDLHDKILHVLFDNAFFYVSKSFKSSIL
P81909_DmOR22a  -FSTFWTGVATCLFMFDVSMETFPFCYLCNMIIDDCQEMSNCLFQSDWTSADRRYKSTLV
                      370  He7  380       390       400       410       420
8Z9A_ApOR5      IMMTRVTIPLKFTVGYIFTINLNLLLKILKMSYTVLNVLLSSET-----
P81909_DmOR22a  YFLHNLQQPITLTAGGVFPISMQTNLAMVKLAFSVVTVIKQFNLAERFQ
```

Figure S4. Final alignment of *Dm*OR22a with *Ap*OR5 (PDB ID:8Z9A)

Pairwise alignment of *Dm*OR22a with *Ap*OR5 (PDB ID:8Z9A) obtained from AlignMe. Helices 1 to 7 are marked in red for the template and in green for the target sequence.

```
                                                                          He1
8Z9A_ApOR5              --------------------IDTINMFLQMTGCTDSKAMLYLTYFEFLITFYYLIATYAS
A0A7L8XZ66_ItOR46       MNAFPDSDSLTALKFTSVLGLFP----WKLAFQQNKFYQTLYYWYSLFVLLWDIGFVFTS

                                  70        80        90   He2  100       110       120
8Z9A_ApOR5              IVHFEQS-------VTIQLFALLCMLIECVILLNITFRLYHKNHIREMHQYSR-----RL
A0A7L8XZ66_ItOR46       YVELVILLRGEVLHIDEICTNIRITTIYTCIIIRLVMIRTSSGLLKLIQEIIDSDKQVTI

                                 130       140  He3  150       160       170       180
8Z9A_ApOR5              GIPDSYRSVINVITKYHLIASNIFVVFPVTYAIFCDSVR------------VGDPFTFPF
A0A7L8XZ66_ItOR46       VDDEETTKLVKTETSINNLKFIWYVGICCSIGLQFFIRPLVAEPEIIQIGNITEVGPRDL

                                 190       200  He4  210       220       230       240
8Z9A_ApOR5              LDVLPMHTDNLAIYACKYLVYAISVYIAHVELCFINTTFIYYVGVLKHRLETIVQTIGEA
A0A7L8XZ66_ItOR46       IILTWFPFDEQKYYWVAYFLQVIDGIIGTLFVALSDVFIVNLILYPTTQLKKLQHIFRNF

                                 250       260  He5  270       280       290       300
8Z9A_ApOR5              FA-----------DN----DEQKFKYAIIQHQKLLSYFNTMKIVFSKPILLSMSFNAIYF
A0A7L8XZ66_ItOR46       EHYQQSYKRLNSCETEESAGIKVITHLVQRHQRIIKFVDTFNGWMGPLMVFDFLQSSIQI

                                 310       320  He6  330       340       350       360
8Z9A_ApOR5              GLTTSFVIQAIRGYINQAILSICIASSAAAVINITIYTFYGSELMDLHDKILHVLFDNAF
A0A7L8XZ66_ItOR46       ASILISDLR---RDITFAMVCFIVTFFVGMVLRLYLHYYSANELILESEKLADAIWYSNW

                                 370       380       390  He7  400        410       420
8Z9A_ApOR5              FYVSKSFKSSILIMMTRVTIPLKFTVGYIFTINLNLLLKILKMSYTVLNVLLSSET
A0A7L8XZ66_ItOR46       YEQSPKIKYLMLIVIIRAQKSLKYNIGAFGIMSLESGIAILKATYTYITVFTSNN-
```

Figure S5. Final alignment of *It*OR46 with *Ap*OR5 (PDB ID:8Z9A)

Pairwise alignment of *It*OR46 with *Ap*OR5 (PDB ID:8Z9A) obtained from AlignMe. Helices 1 to 7 are marked in red for the template and in green for the target sequence.

```
                      10        20        30        40  He1   50        60
7LIG_MhOR5      ----DDYIHLRKWIKRIGIILRISGHWPFRLPHEKRNQHKSKFRQVYSCLVITLGFITCS
J7FCG8_OfOR3    MPAVHQNPSTLSYIITVKNALGPSGIWPSNIFEDKLQPLFFRIHRETLPYHTMLIVFGGL
                      70        80        90 He2    100       110       120
7LIG_MhOR5      CYCIGLCLS-ESIAQALNNITVTSYFLQSCVCYVSFIINSRKLETLFNYLFENEVVGCPR
J7FCG8_OfOR3    YYLSDNFRIMSFLDMGHIILSTFLAMVTAMRSVVPNLKIYVALLTKLGREIHLMHFAHKG
                      130       140 He3   150       160       170       180
7LIG_MhOR5      GYKMSSIKTTLFRCKFVAFSLGILSFFGWLMWTLLPLAVLV---VDQTSLRFVEAW----
J7FCG8_OfOR3    PYYEEINKTVDKASHIYTKFIVVFMYMTMMMFNITPIYNISKNILSSKTENSTQEYALYY
                      190 He4   200       210       220       230       240
7LIG_MhOR5      -YPFDTTTSPMNEVIAIYEAVAMIFLITAPMSSDIMFCVLMIFIVEHLKCLGMAIECTLK
J7FCG8_OfOR3    SFPGINPMN-YYPTTTVYNFYLSYNCGIMMCGLDLVLFLMIFQLIGHVYILRHNLENFPS
                      250       260       270       280 He5   290       300
7LIG_MhOR5      GD--------------------------------ATSLCNIVDSHVKIYRTMEIVQSVYS
J7FCG8_OfOR3    PKNKVVLNIGDLPRYKNKENCIVEMFDAKENEEVRVRLAECIEHHKIIIRFTDEISVVFG
                      310       320       330       340 He6   350       360
7LIG_MhOR5      SYFATLFFTSCLAVCALAYFLAATSTSFTRVPGMVLYLMYIFLRIFLLCLLATEVAEQGL
J7FCG8_OfOR3    PILAFNYMFHMVGCCLLLLECSA----GNQIIRYGPLTTVVFGQLIQISVMFEMLGAETE
                      370       380       390 He7 400       410       420
7LIG_MhOR5      NLCHAGYSSKLVLASDHVRSTIQAIATRAQIPLSITGARFFTVNLSFLASMAGVMLTYFI
J7FCG8_OfOR3    KLKDSAYFVPWECMNISNRRTAHIMLHKMQDKISIKALGLAAVGVNTMMGILKTTFSYYA

7LIG_MhOR5      VLLQVN-
J7FCG8_OfOR3    FLQTMGD
```

Figure S6. Final alignment of *Of*OR3 with *Mh*OR5 (PDB ID:7LIG)

Pairwise alignment of *Of*OR3 with *Mh*OR5 (PDB ID:7LIG) obtained from AlignMe. Helices 1 to 7 are marked in red for the template and in green for the target sequence.

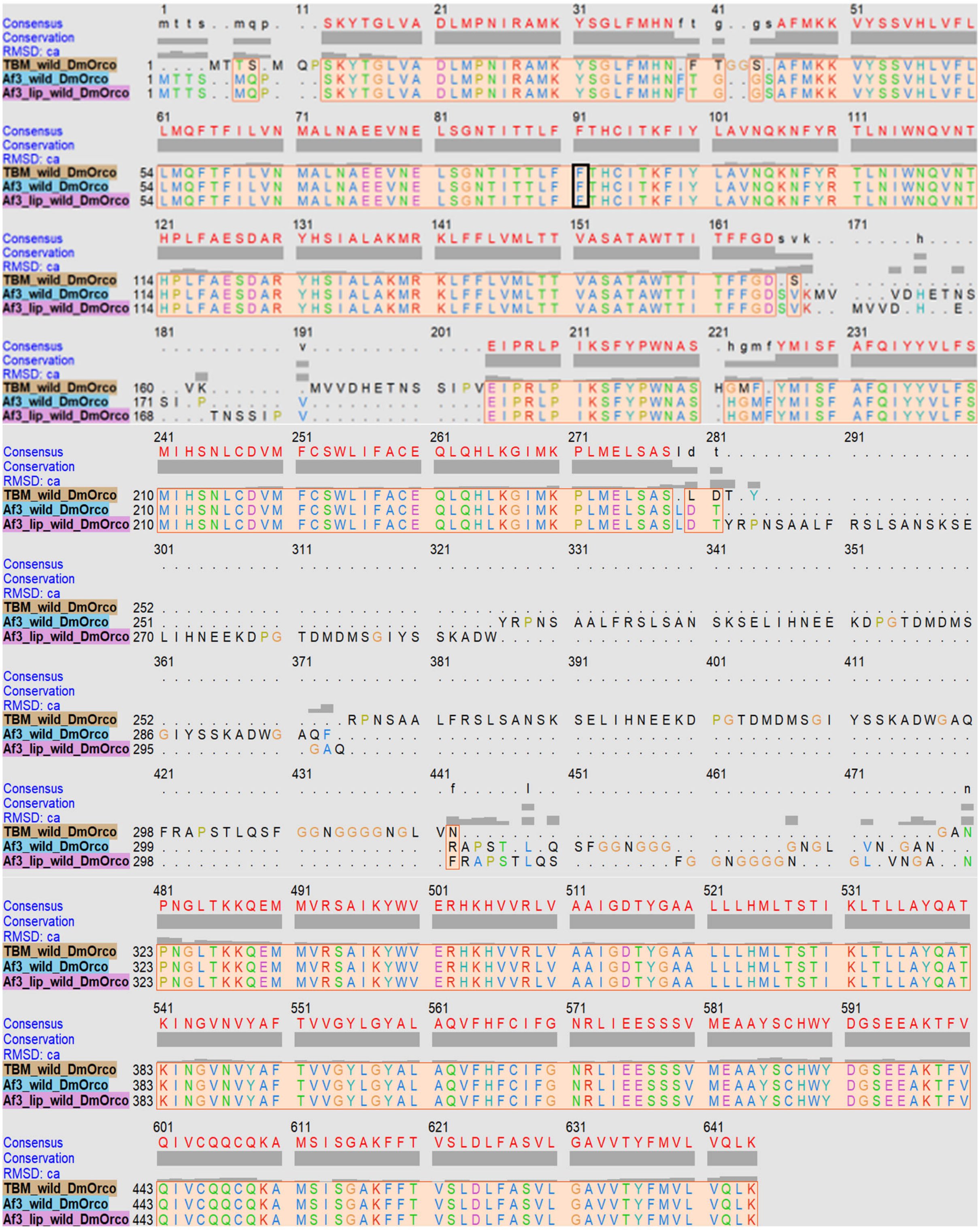

Figure S7. Sequence alignment of the predicted *Dm*Orco models derived from structural superposition. The mutagenesis evidence site (Phe 84) is marked in black.

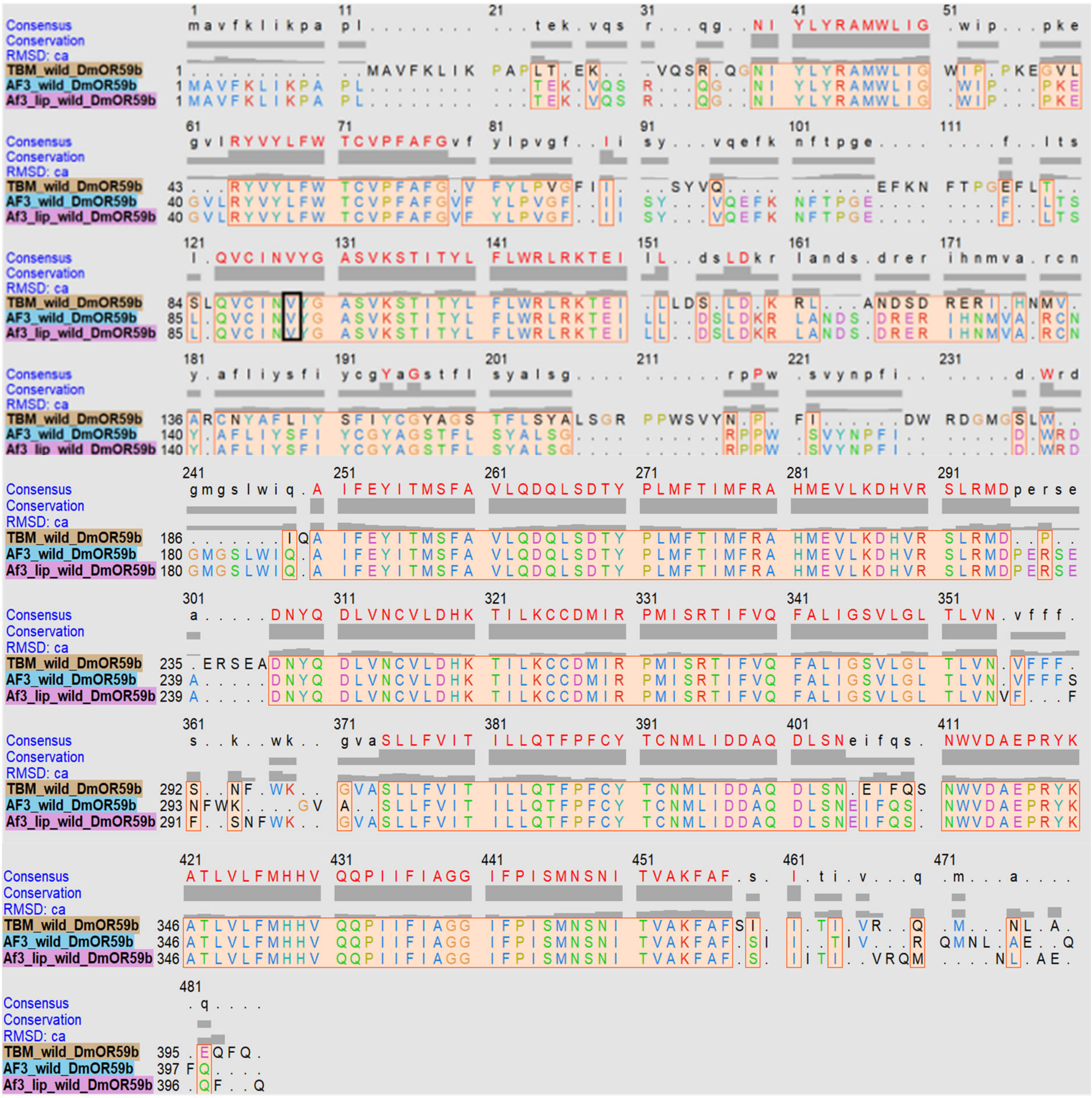

Figure S8. Sequence alignment of the predicted *Dm*OR59b models derived from structural superposition. The mutagenesis evidence site (Val 91) is marked in black.

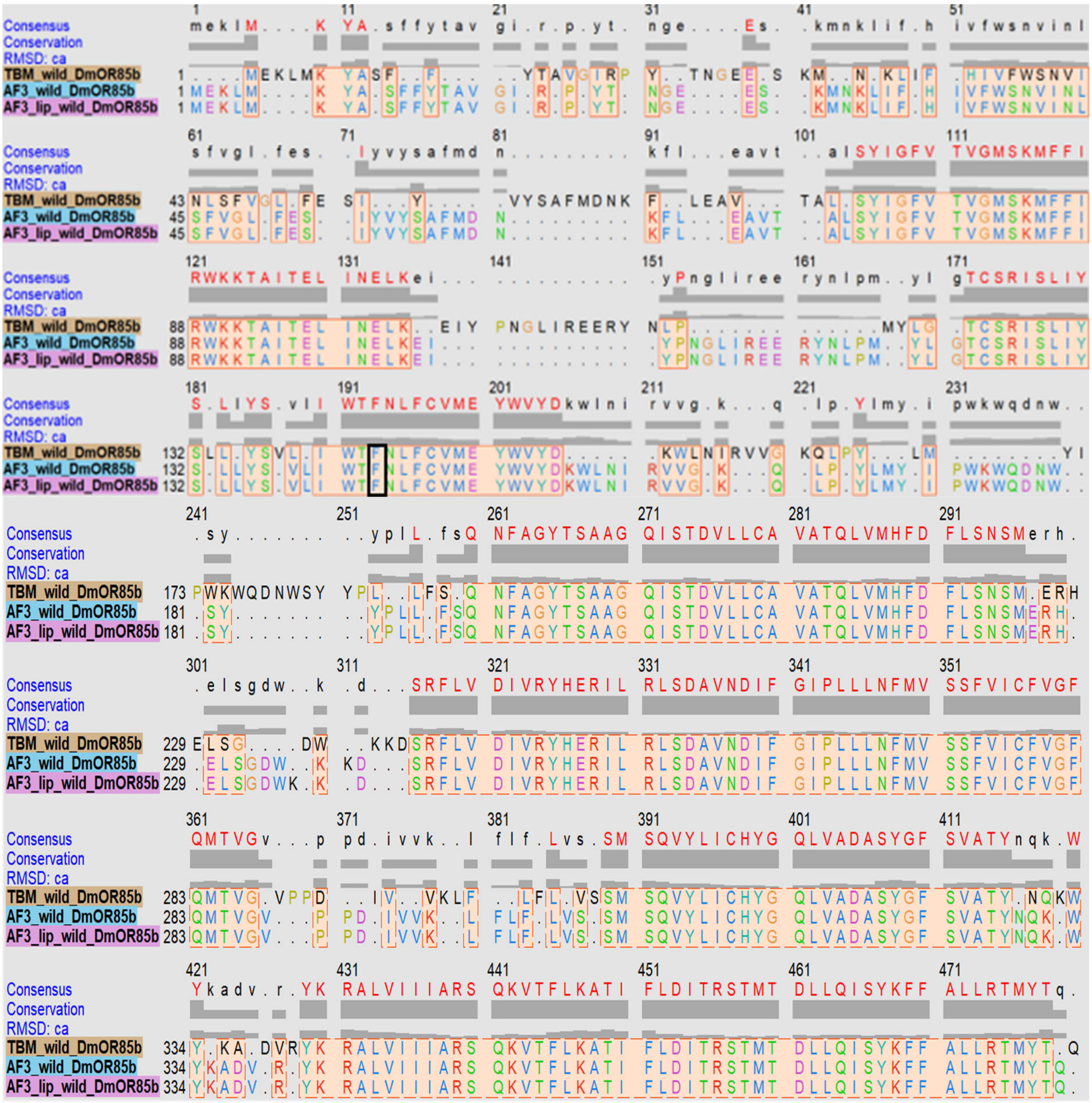

Figure S9. Sequence alignment of the predicted *Dm*OR85b models derived from structural superposition. The mutagenesis evidence site (Phe 142) is marked in black.

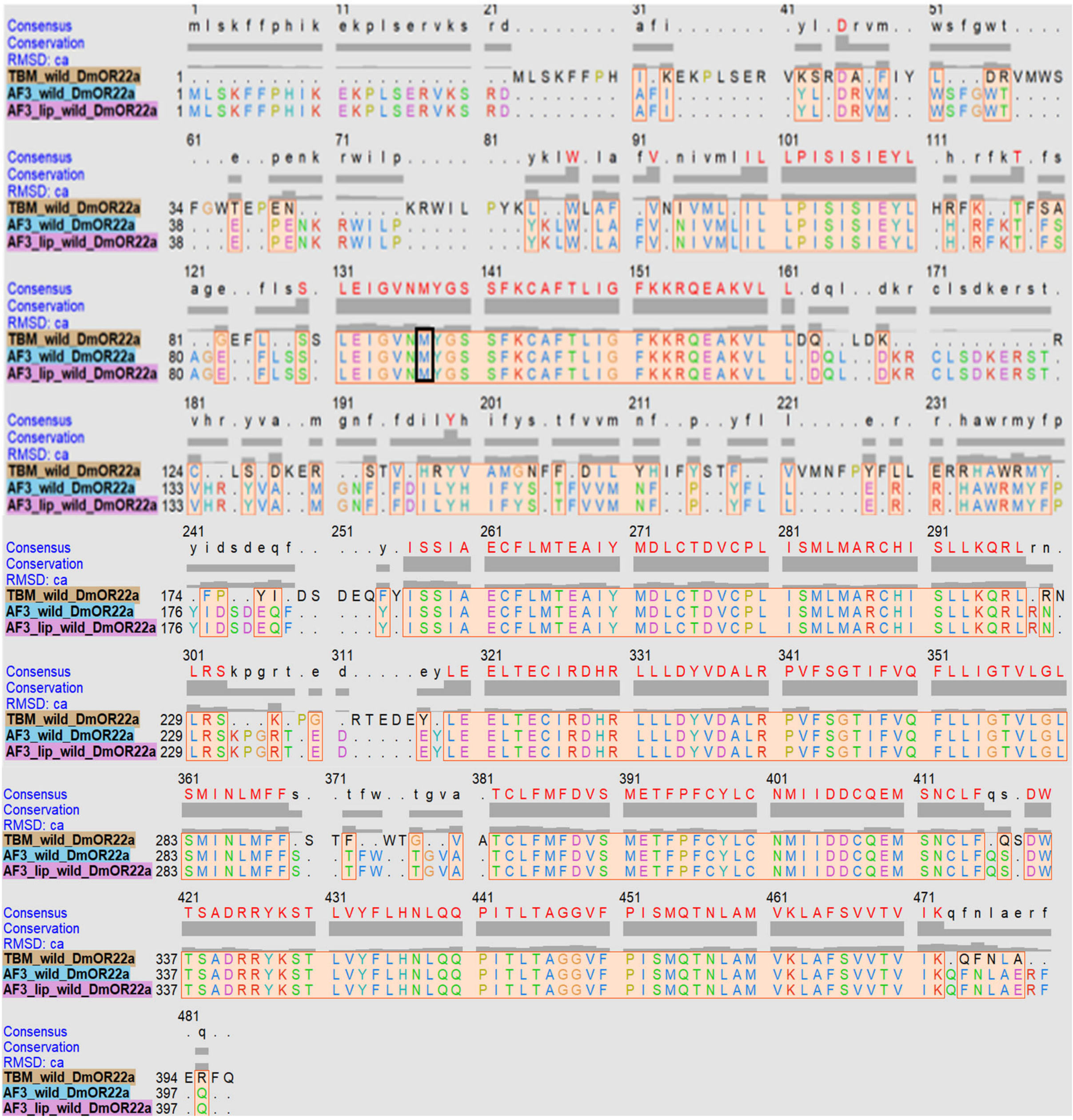

Figure S10. Sequence alignment of the predicted of *Dm*OR22a models derived from structural superposition. The mutagenesis evidence site (Met 93) is marked in black.

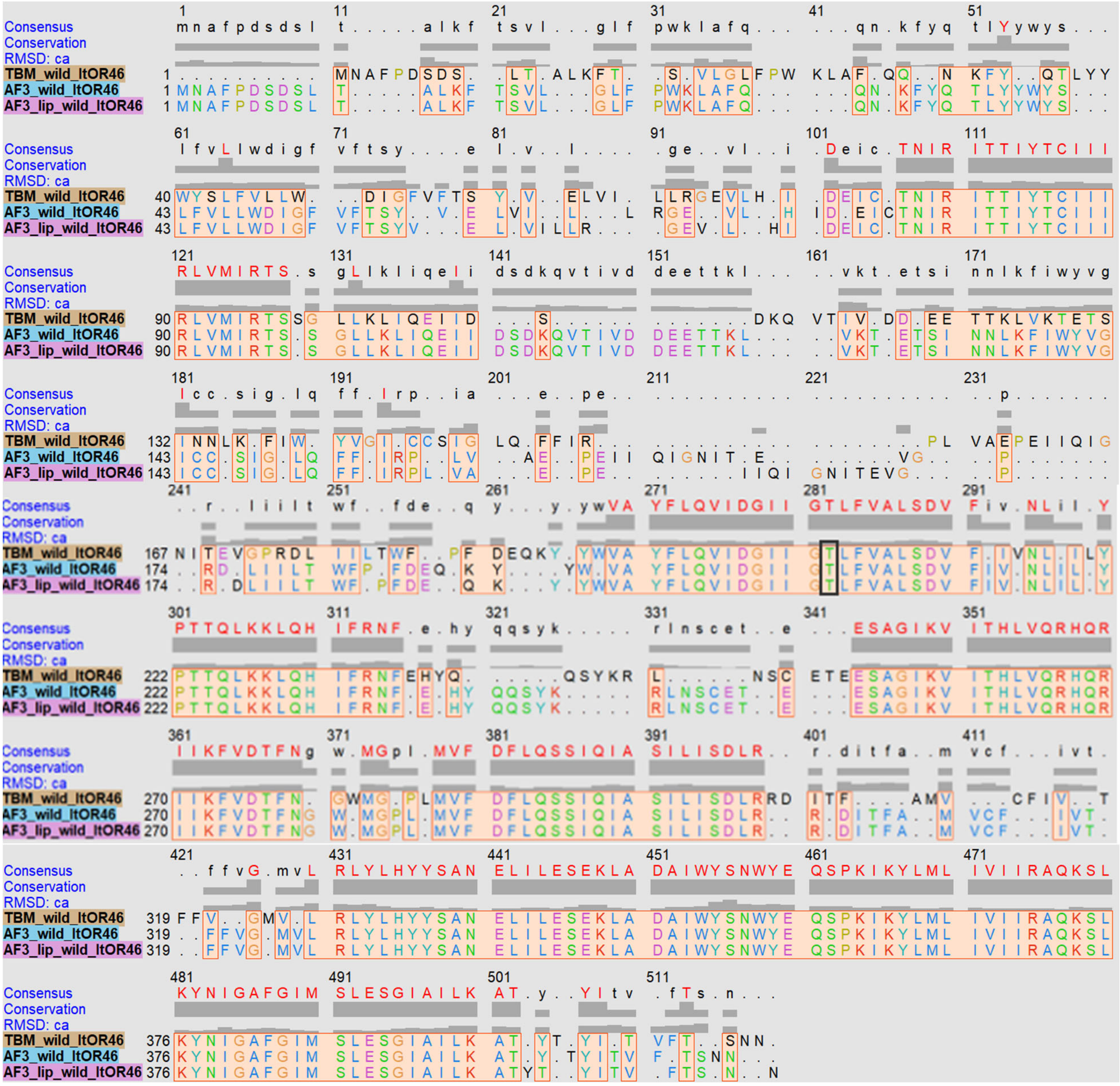

Figure S11. Sequence alignment of the predicted *It*OR46 models derived from structural superposition. The mutagenesis evidence site (Thr 205) is marked in black.

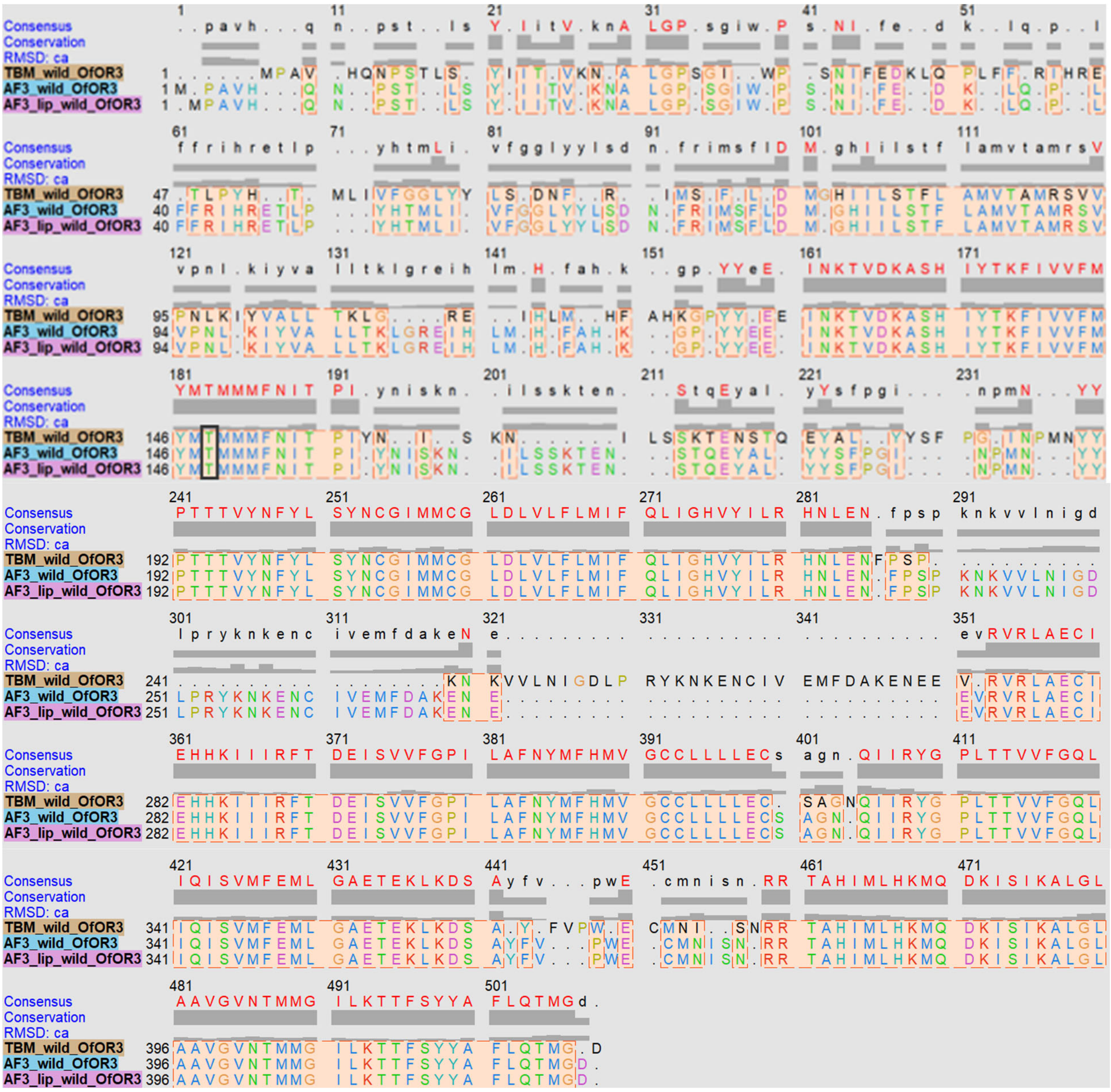

Figure S12. Sequence alignment of the predicted *Of*OR3 models derived from structural superposition. The mutagenesis evidence site (Thr 148) is marked in black.

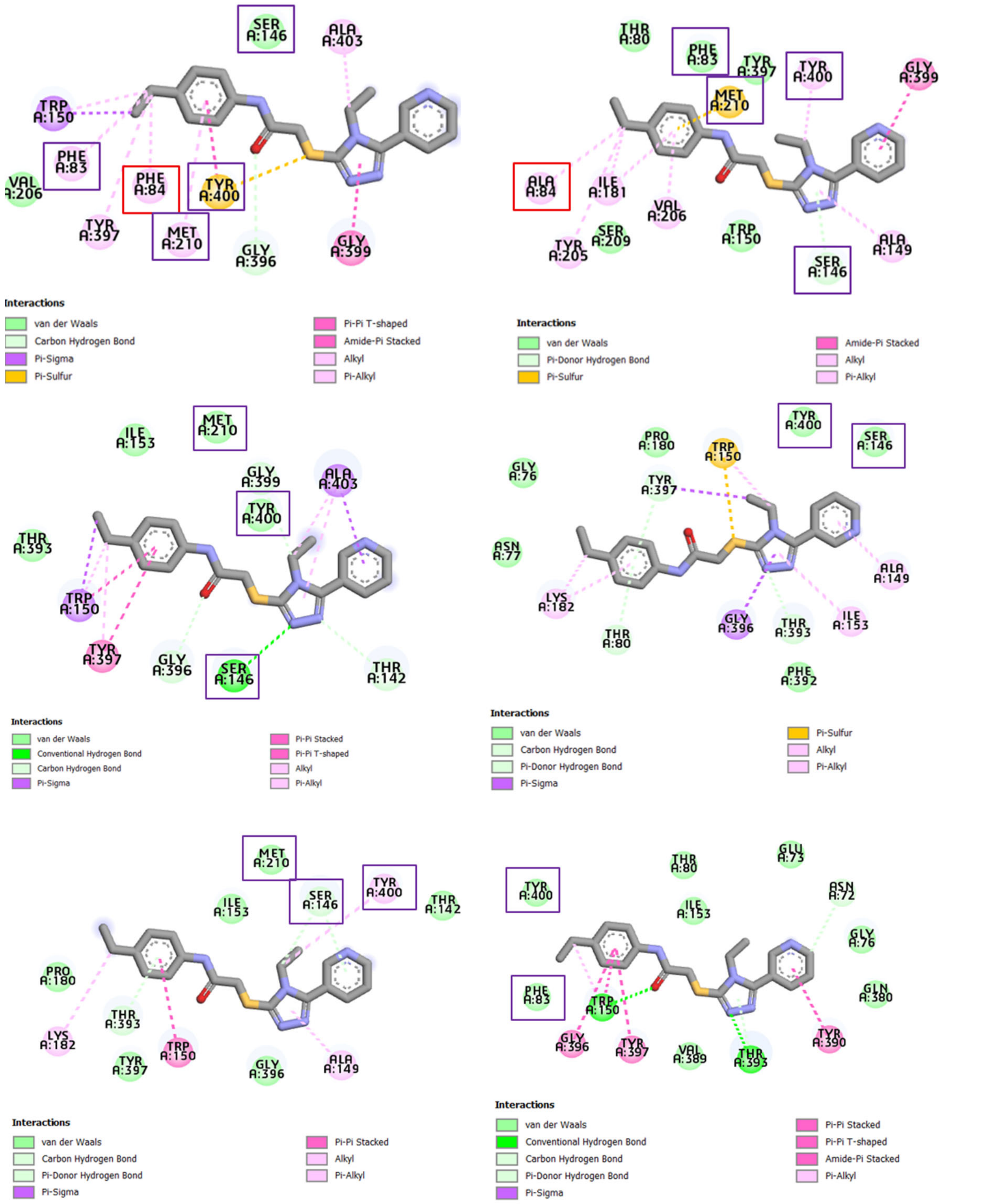

Figure S13. 2D view of the binding pocket interactions for predicted models of *Dm*Orco with VUAA1

Interacting residues shown for (a) *Wt*TBM, (b) *Mt*TBM, (c) *Wt*AF3, (d) *Mt*AF3, (e) *Wt*AF3_lip, and (f) *Mt*AF3_lip complexes. The ligand binding residue 84, having mutagenesis data for F84A, is boxed in red. The five conserved binding pocket residues as reported [31] are boxed in purple and red.

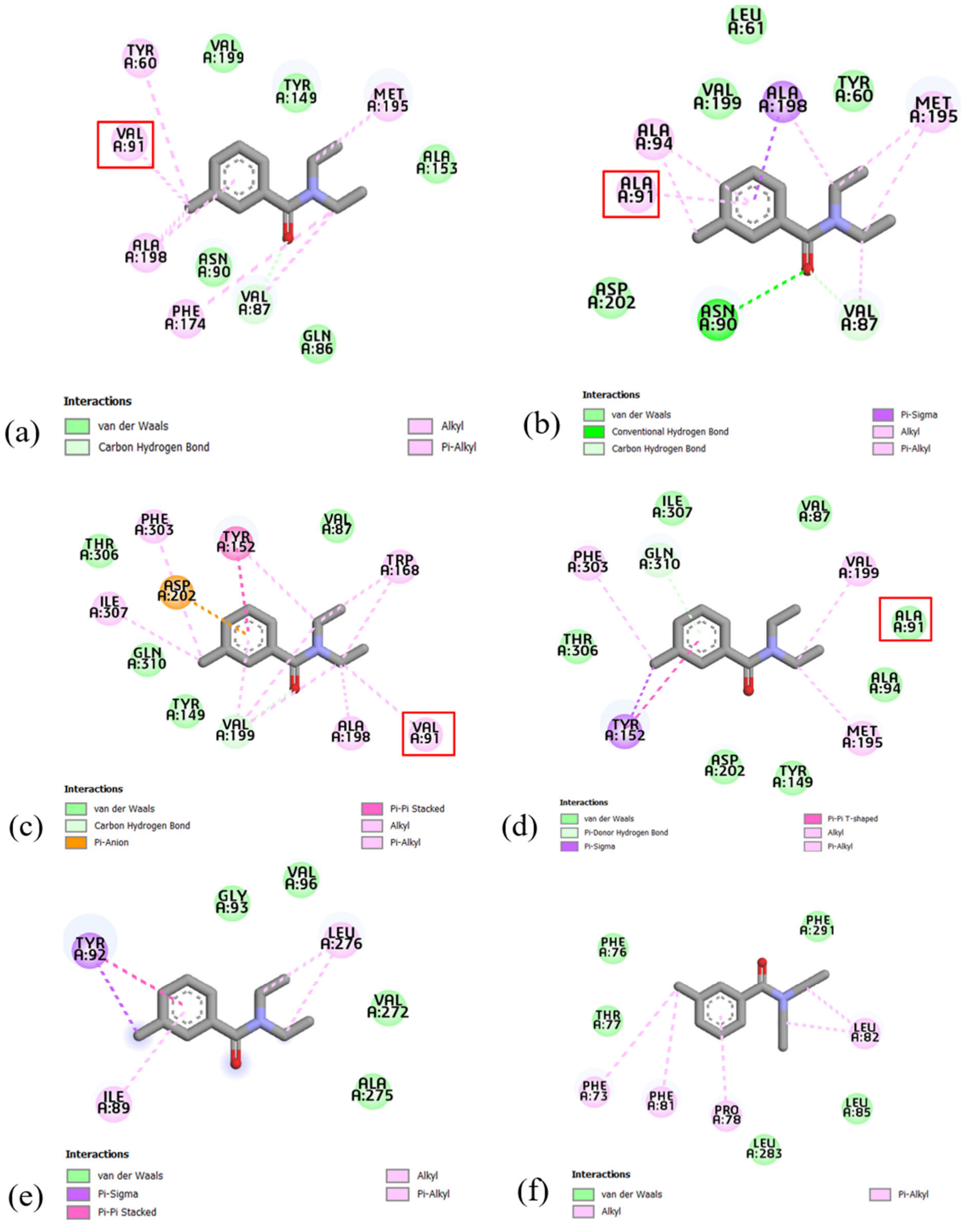

Figure S14. 2D view of the binding pocket interactions for predicted models of *Dm*OR59b with DEET.

Interacting residues shown for (a) *Wt*TBM, (b) *Mt*TBM, (c) *Wt*AF3, (d) *Mt*AF3, (e) *Wt*AF3_lip, and (f) *Mt*AF3_lip complexes. The ligand binding residue 91, having mutagenesis data for V91A, is boxed in red.

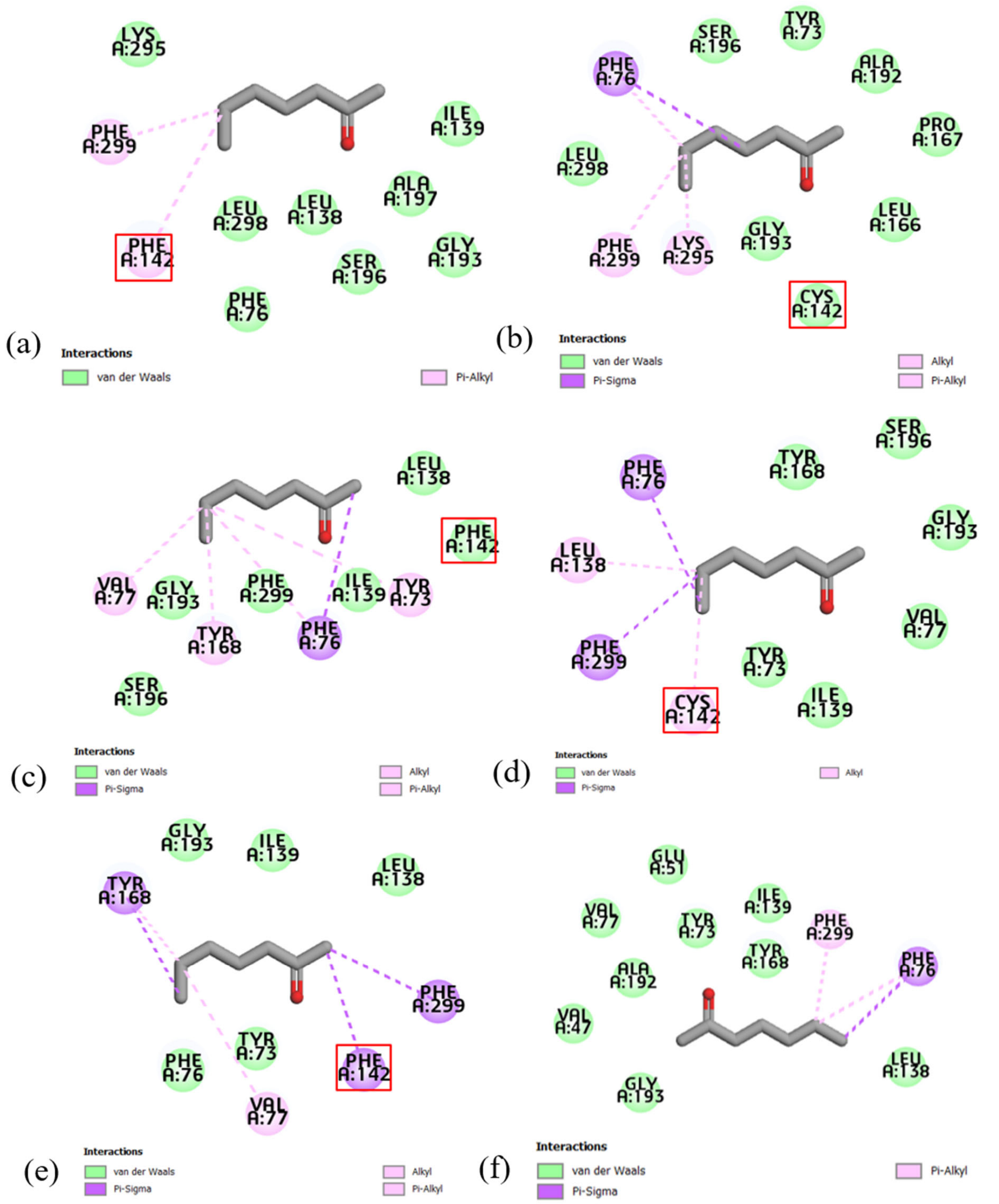

Figure S15. 2D view of the binding pocket interactions for predicted models of *Dm*OR85b with 2-heptanone.

Interacting residues shown for (a) *Wt*TBM, (b) *Mt*TBM, (c) *Wt*AF3, (d) *Mt*AF3, (e) *Wt*AF3_lip, and (f) *Mt*AF3_lip complexes. The ligand binding residue 142, having mutagenesis data for F142C, is boxed in red.

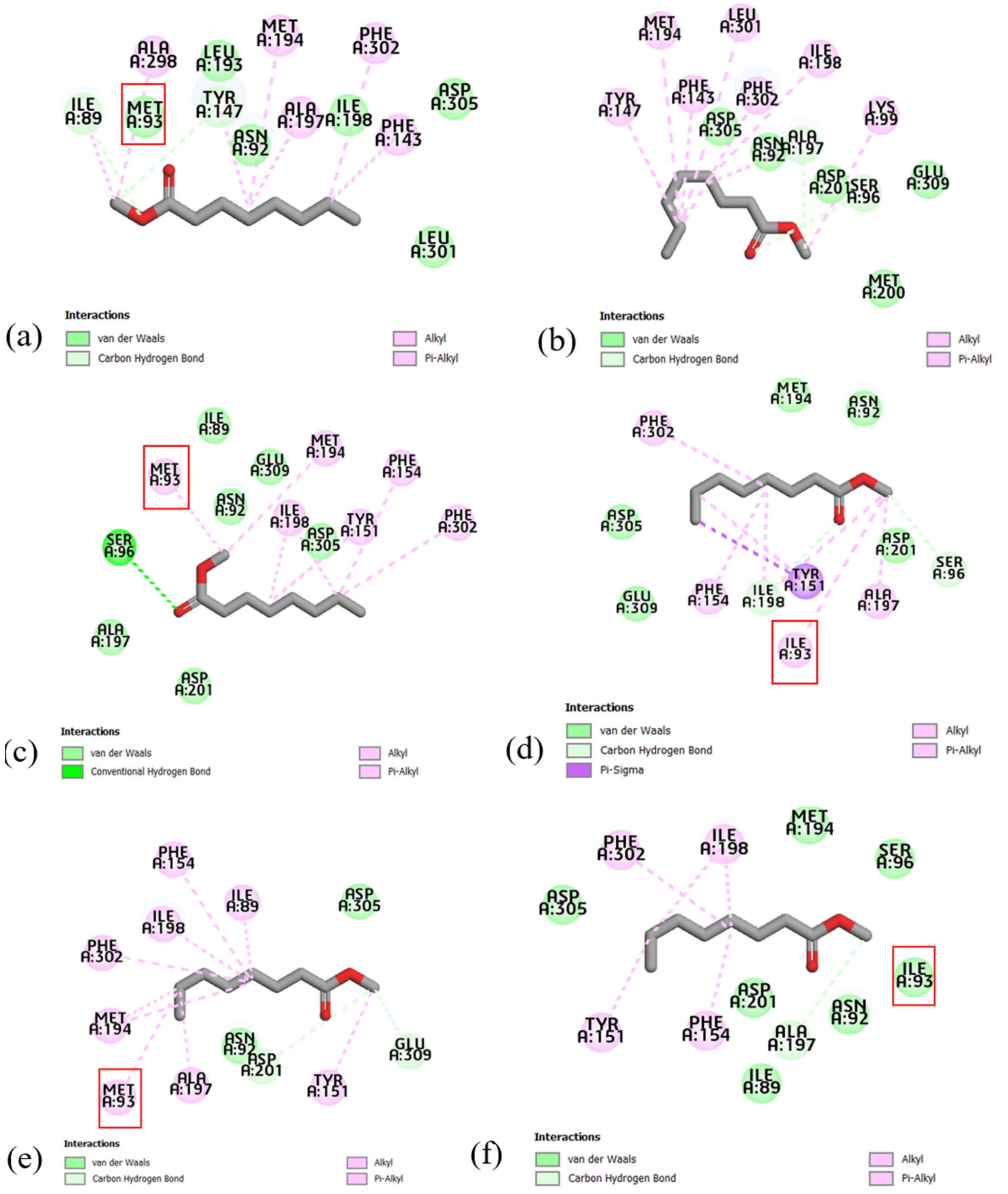

Figure S16. 2D view of the binding pocket interactions for predicted models of *Dm*OR22a with methyl octanoate.

Interacting residues shown for (a) *Wt*TBM, (b) *Mt*TBM, (c) *Wt*AF3, (d) *Mt*AF3, (e) *Wt*AF3_lip, and (f) *Mt*AF3_lip complexes. The ligand binding residue 93, having mutagenesis data for M93I is boxed in red.

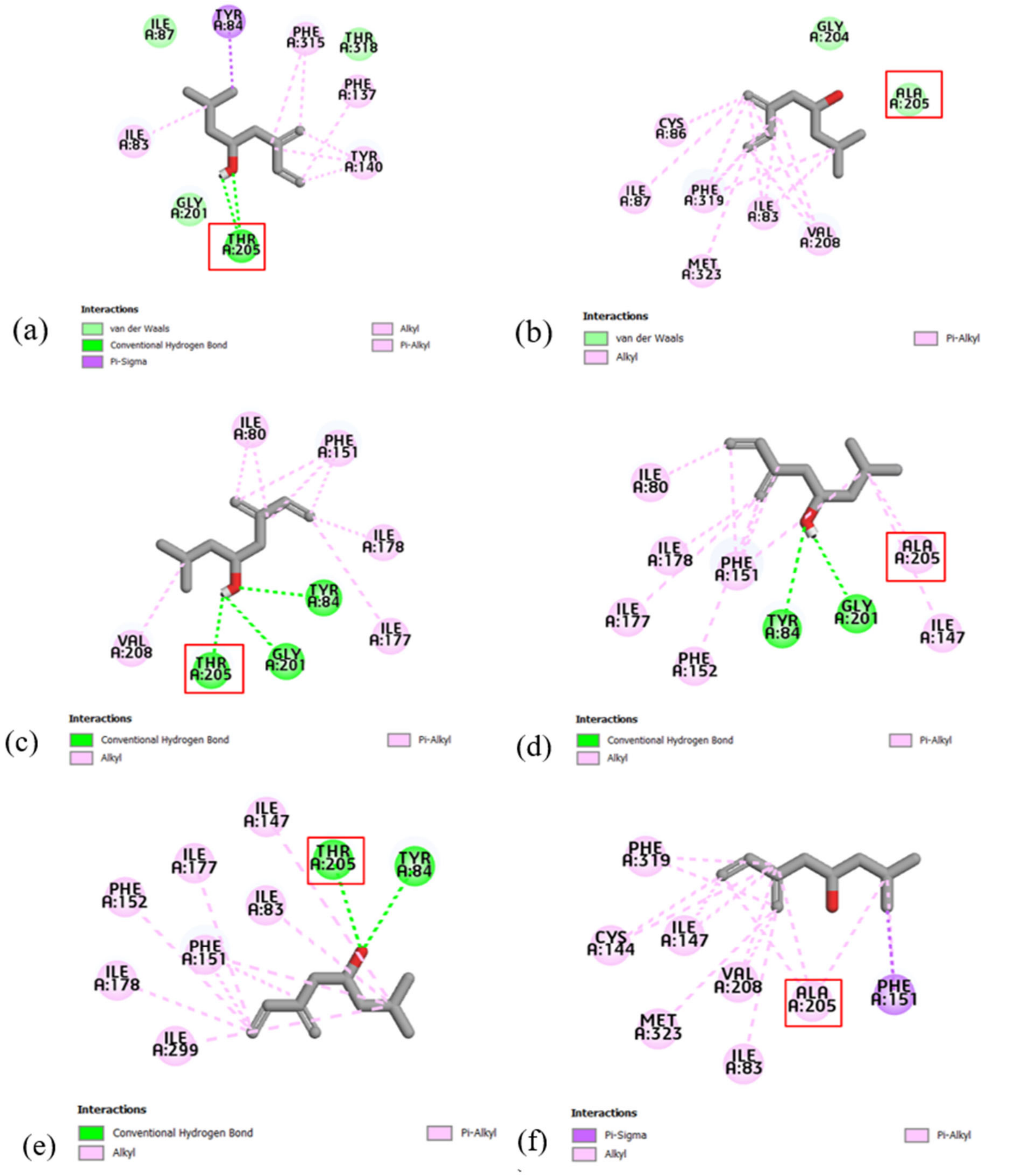

Figure S17. 2D view of the binding pocket interactions for predicted models of *It*OR46 with (*S*)-(−)-ipsenol.

Interacting residues shown for (a) *Wt*TBM, (b) *Mt*TBM, (c) *Wt*AF3, (d) *Mt*AF3, (e) *Wt*AF3_lip, and (f) *Mt*AF3_lip complexes. Residue 205, having mutagenesis data for T205A, is boxed in red.

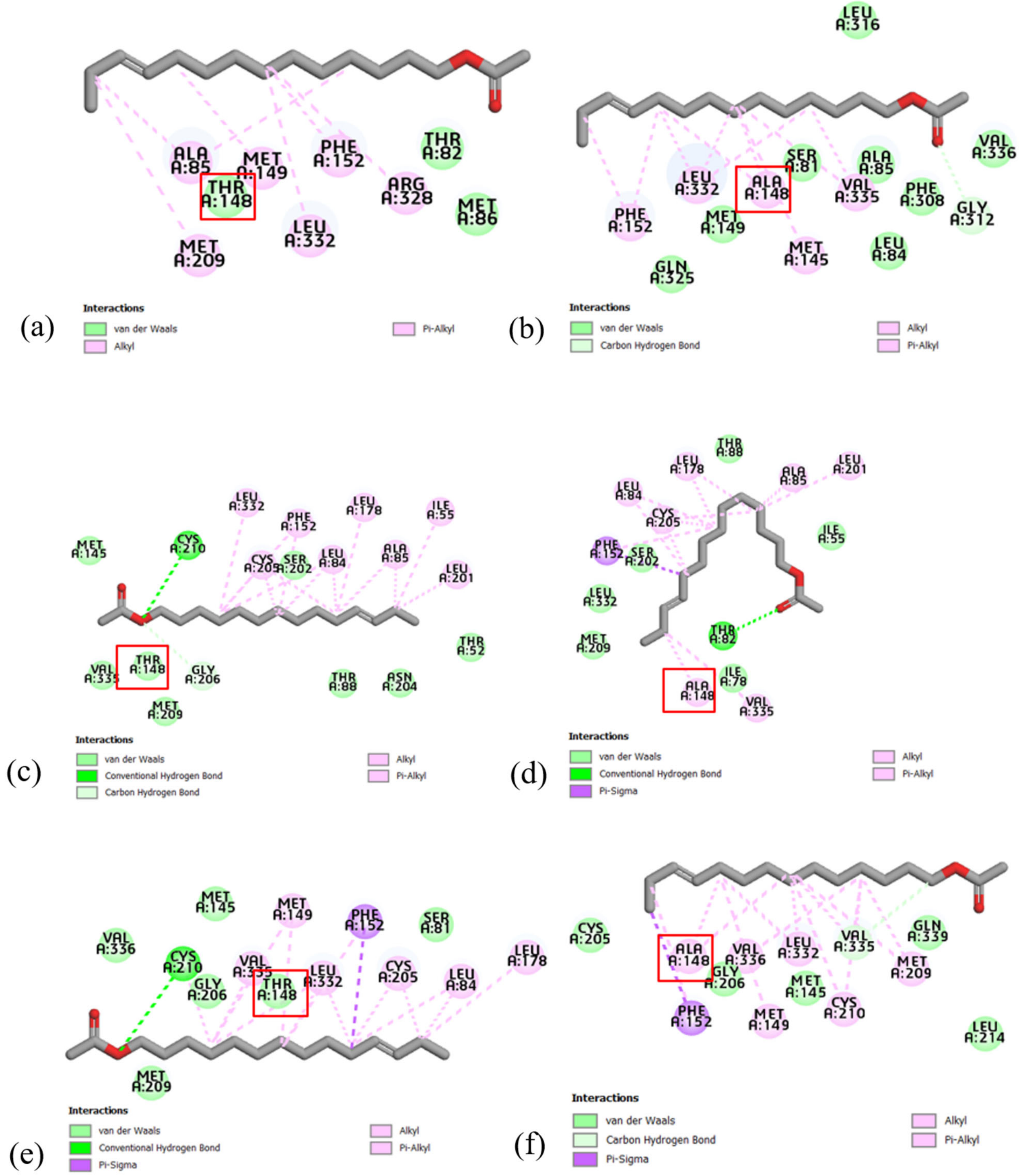

Figure S18. 2D view of the binding pocket interactions for predicted models of *Of*OR3 with (E)-11-tetradecenyl acetate.
Interacting residues shown for (a) *Wt*TBM, (b) *Mt*TBM, (c) *Wt*AF3, (d) *Mt*AF3, (e) *Wt*AF3_lip, and (f) *Mt*AF3_lip complexes. Residue 148, having mutagenesis data for T148A, is boxed in red.

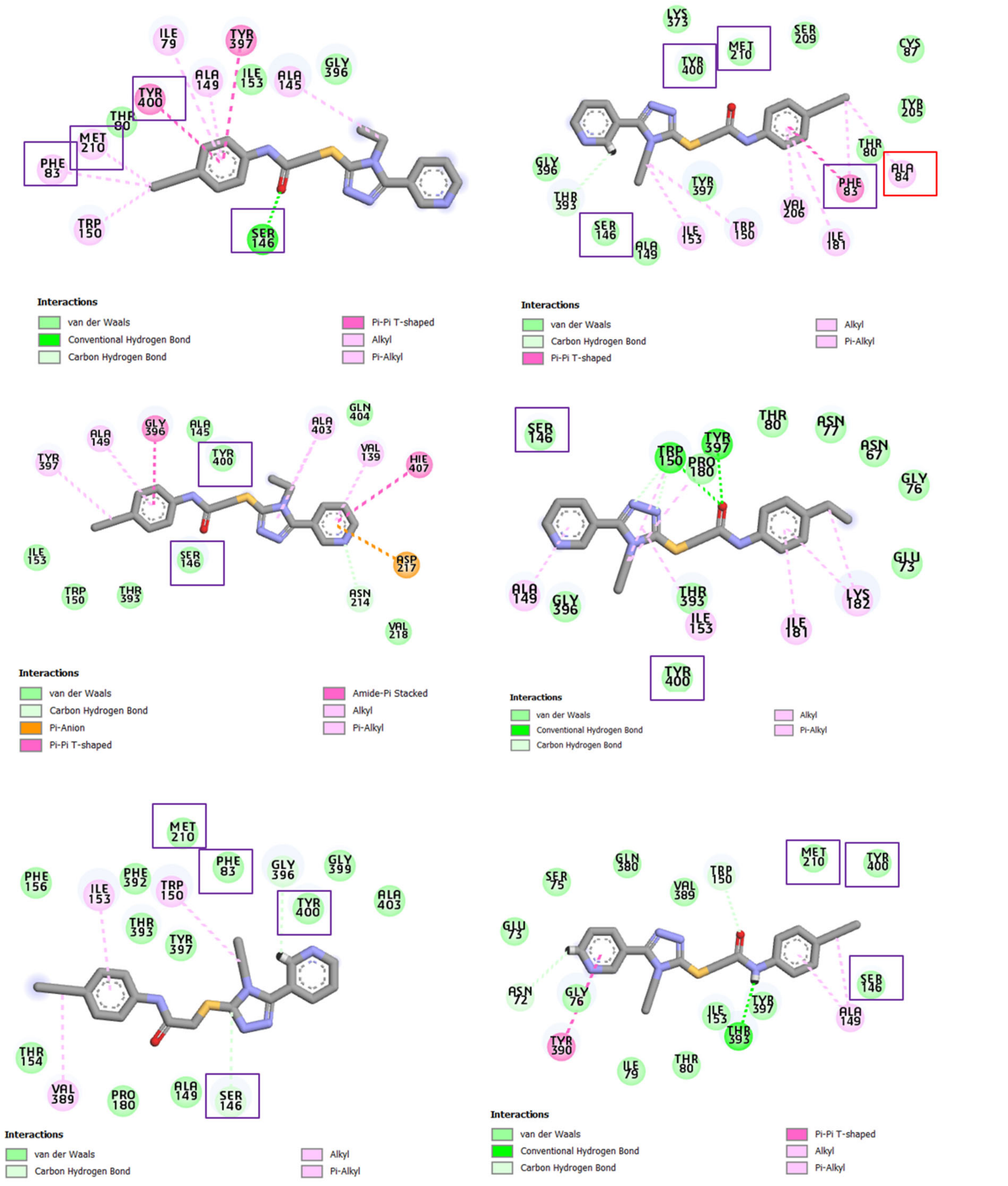

Figure S19. Interactions of VUAA1 after local refinement with all predicted models of *Dm*Orco.

2D view of the interactions of the predicted models of *Dm*Orco with VUAA1 after local refinement for (a) *Wt*TBM [34], (b) *Mt*TBM [34], (c) *Wt*AF3, (d) *Mt*AF3, (e) *Wt*AF3_lip, and (f) *Mt*AF3_lip complexes. Residue 8A having mutagenesis data for F84A, is boxed in red. The binding pocket residues is boxed in purple.

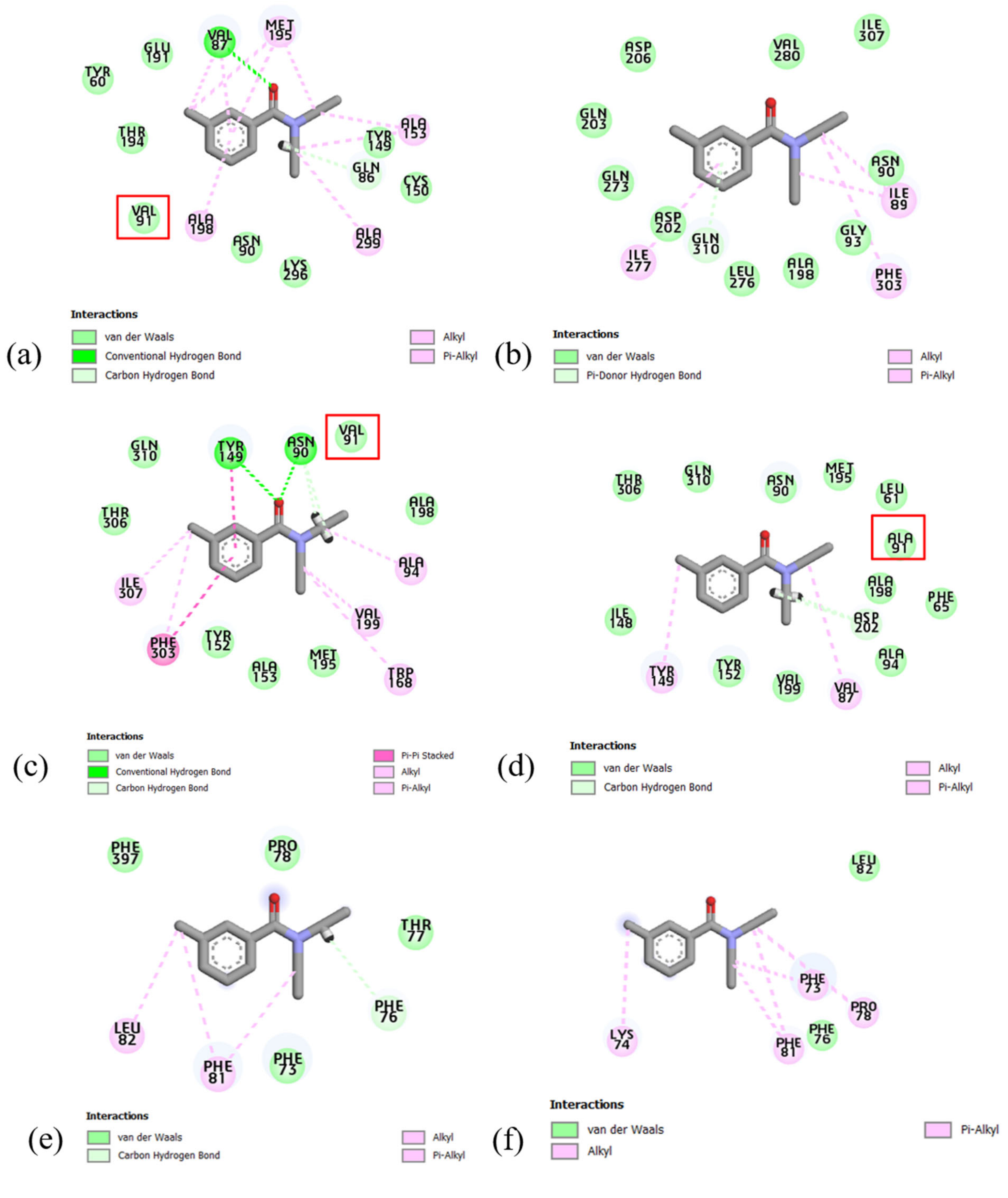

Figure S20. Interactions of DEET after local refinement with all predicted models of *Dm*OR59b.

2D view of the interactions of the predicted models of *Dm*OR59b with DEET after local refinement for (a) *Wt*TBM [34], (b) *Mt*TBM [34], (c) *Wt*AF3, (d) *Mt*AF3, (e) *Wt*AF3_lip, and (f) *Mt*AF3_lip complexes. Residue 91 having mutagenesis data for V91A, is boxed in red.

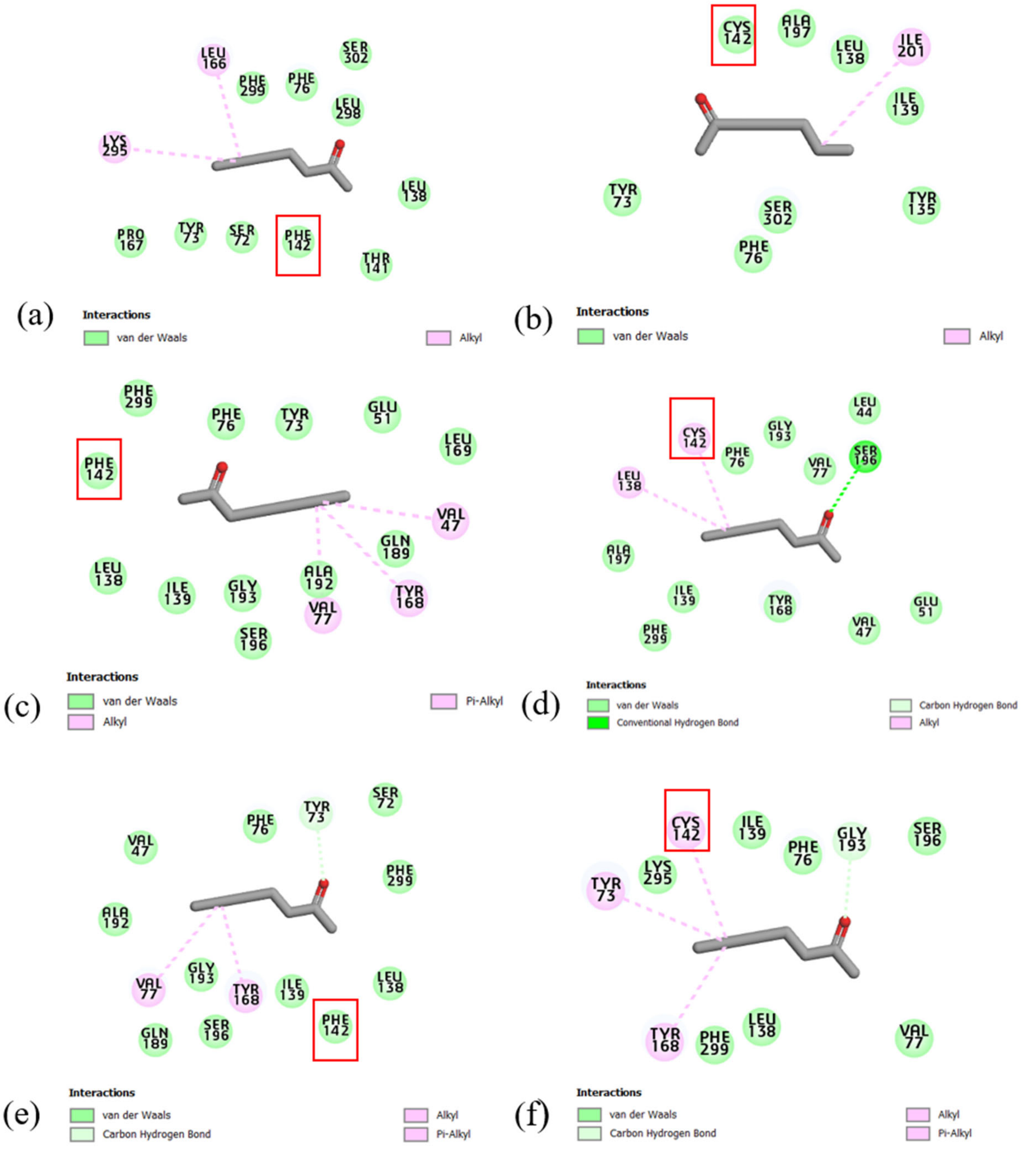

Figure S21. Interactions of 2-heptanone after local refinement with all predicted models of *Dm*OR85b.

2D view of the interactions of the predicted models of *Dm*OR85b with 2-heptanone after local refinement for (a) *Wt*TBM, (b) *Mt*TBM, (c) *Wt*AF3, (d) *Mt*AF3, (e) *Wt*AF3_lip, and (f) *Mt*AF3_lip complexes. Residue 142, having mutagenesis data for F142C, is boxed in red.

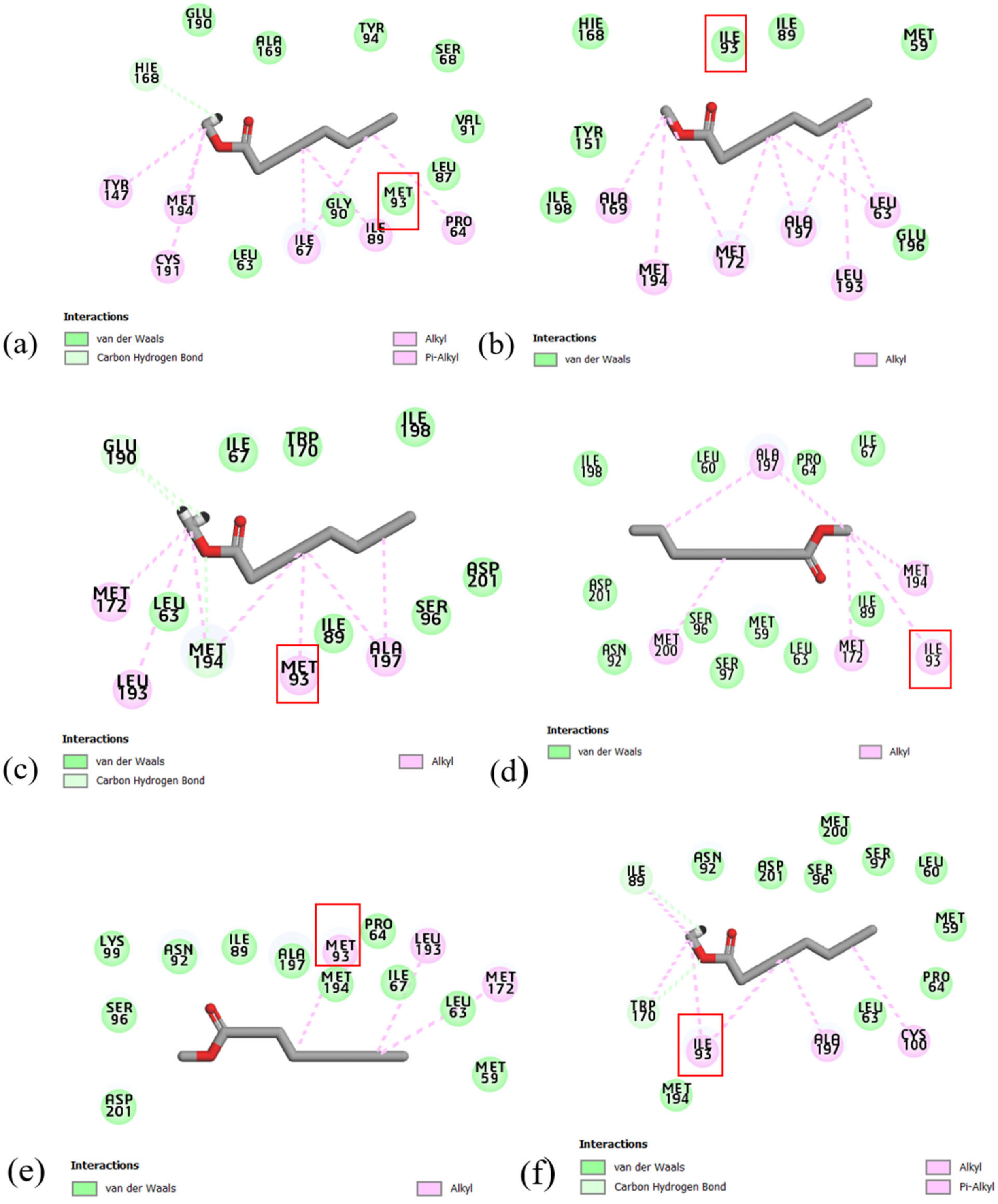

Figure S22. Interactions of methyl octanoate after local refinement with all predicted models of *Dm*OR22a.

2D view of the interactions of the predicted models of *Dm*OR22a with methyl octanoate after local refinement for (a) *Wt*TBM, (b) *Mt*TBM, (c) *Wt*AF3, (d) *Mt*AF3, (e) *Wt*AF3_lip, and (f) *Mt*AF3_lip complexes. Residue 93, having mutagenesis data for M93I, is boxed in red.

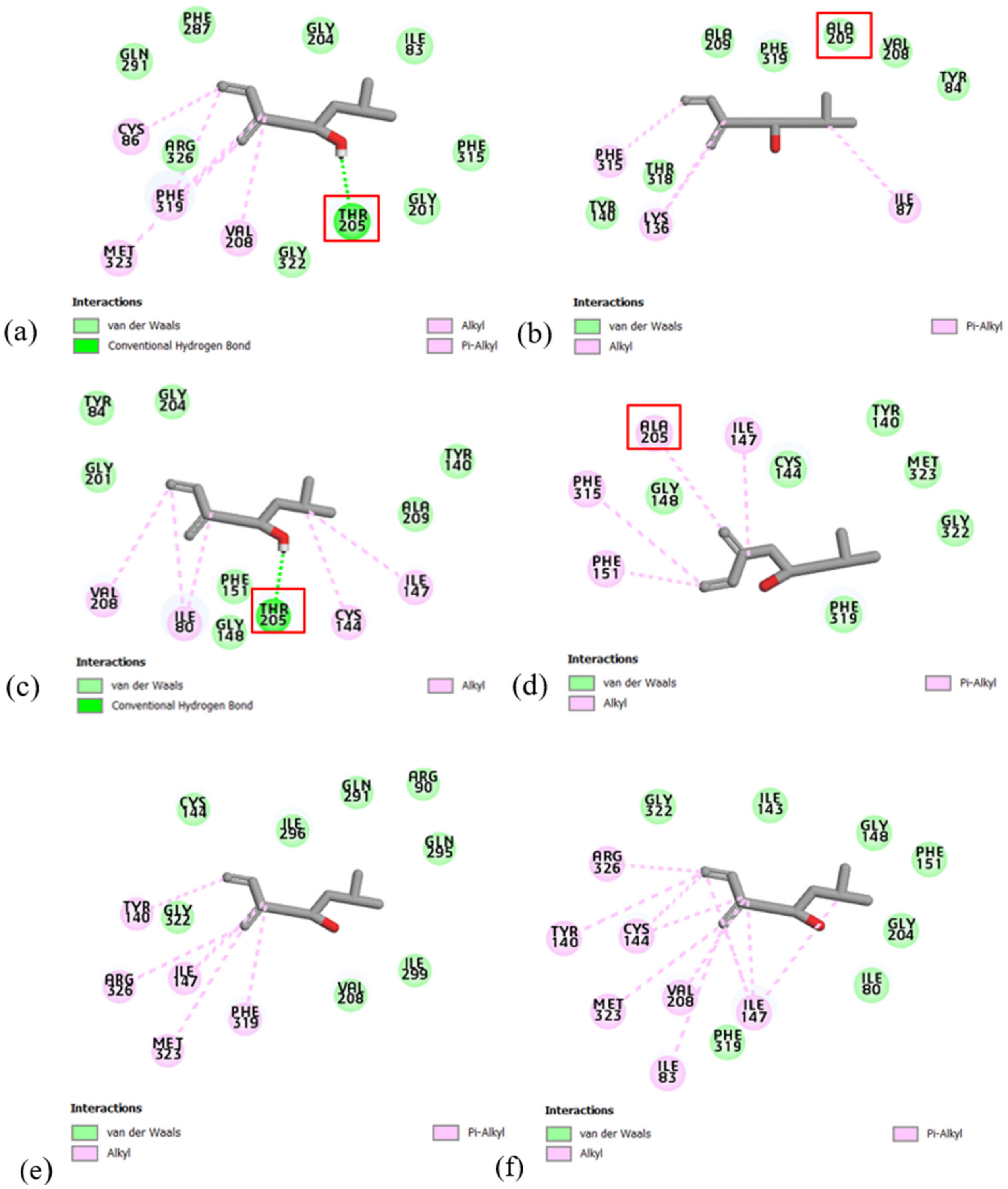

Figure S23. Interactions of (*S*)-(−)-ipsenol after local refinement with all predicted models of *It*OR46.
2D view of the interactions of the predicted models of *It*OR46 with (S)-(−)-ipsenol after local refinement for (a) *Wt*TBM, (b) *Mt*TBM, (c) *Wt*AF3, (d) *Mt*AF3, (e) *Wt*AF3_lip, and (f) *Mt*AF3_lip complexes. Residue 205, having mutagenesis data (T205A) for *It*OR46, is boxed in red.

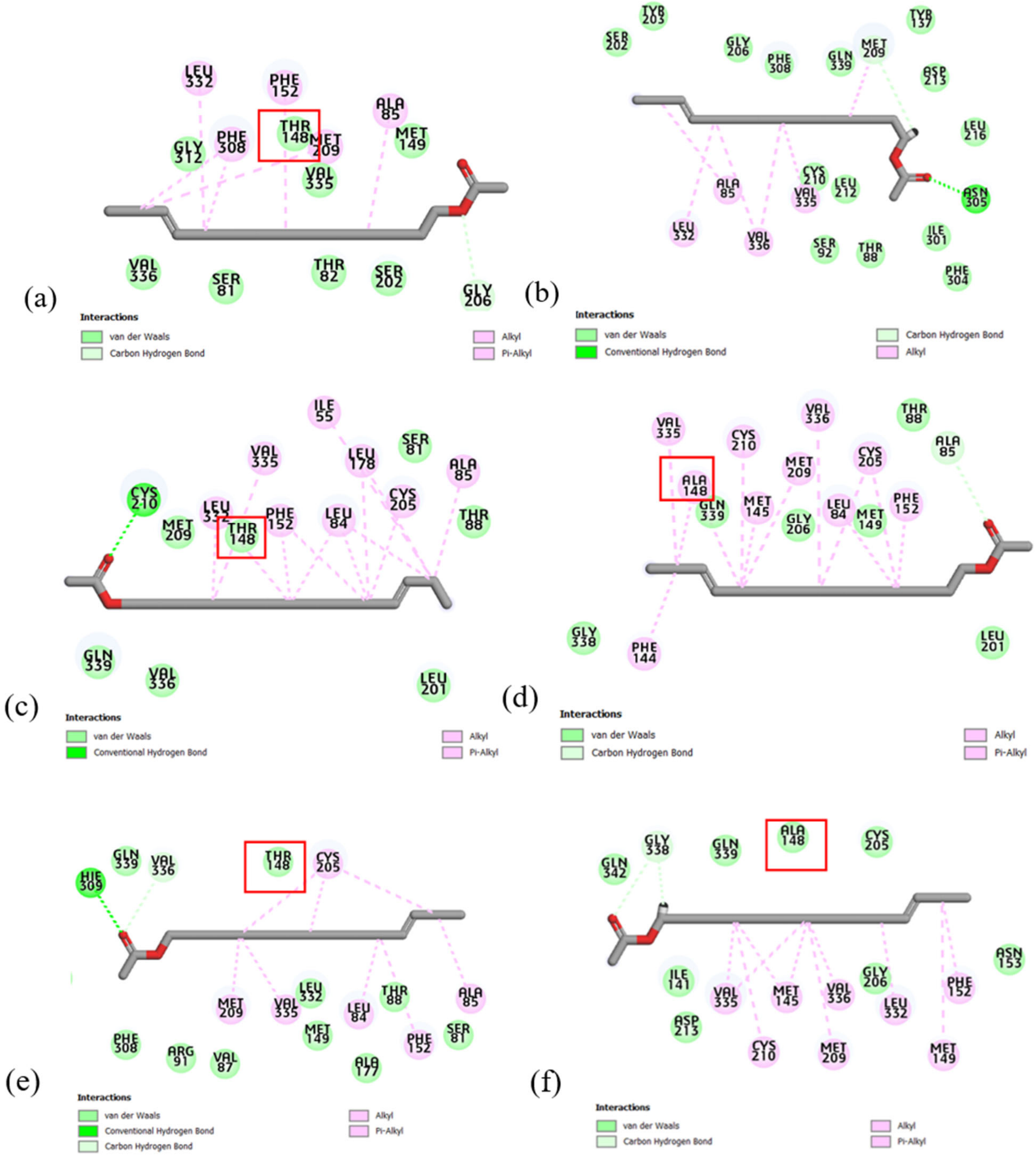

Figure S24. Interactions of (E)-11-tetradecenyl acetate after local refinement with all predicted models of *Of*OR3.

2D view of the interactions of the predicted models of *Of*OR3 with (E)-11-tetradecenyl acetate after local refinement for (a) *Wt*TBM, (b) *Mt*TBM, (c) *Wt*AF3, (d) *Mt*AF3, (e) *Wt*AF3_lip, and (f) *Mt*AF3_lip complexes. Residue 148, having mutagenesis data for T148A, is boxed in red.

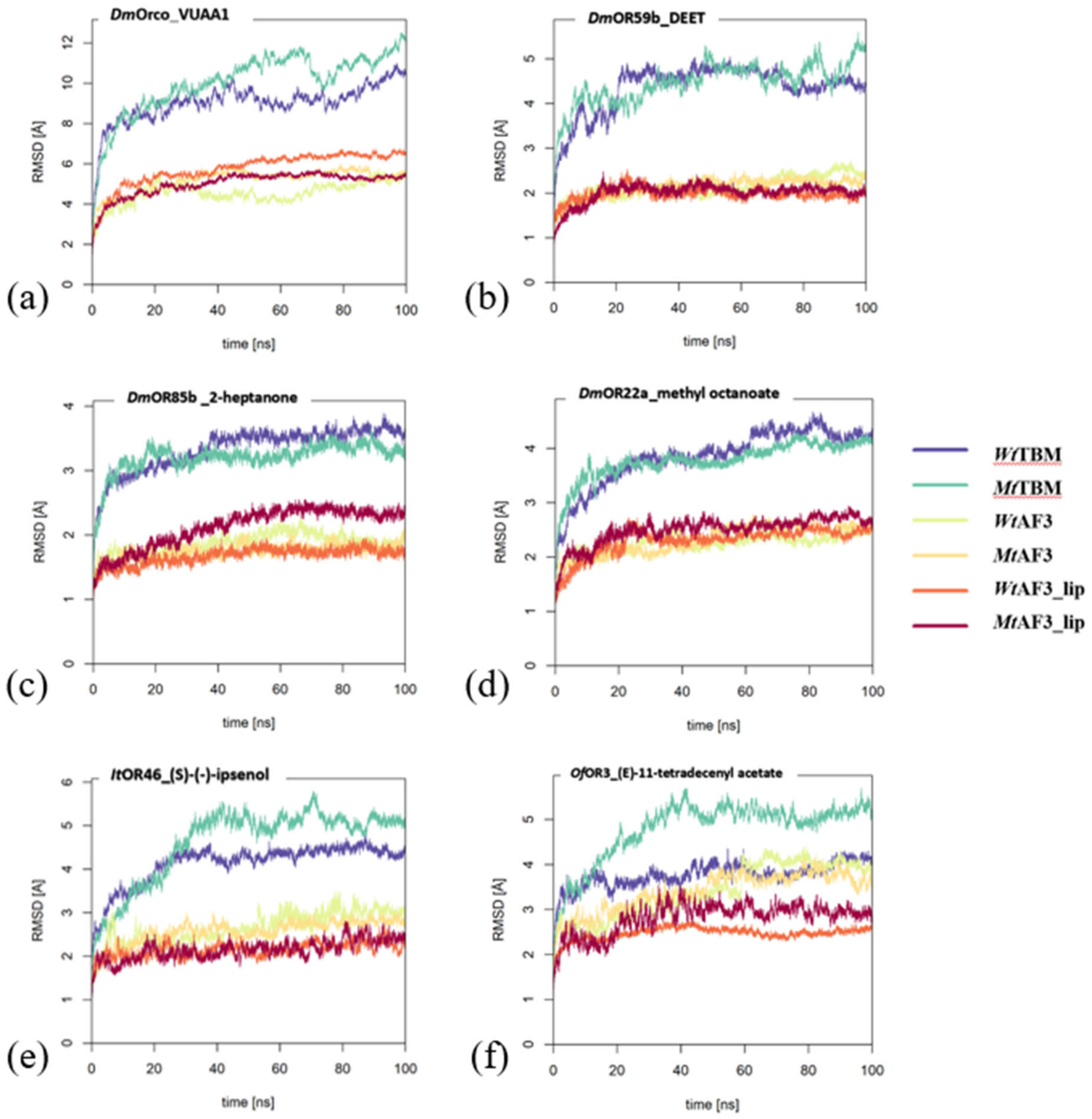

Figure S25. Complex RMSD values of all 36 complexes over the last 100 ns.
Average RMSD values of three replicates shown for the following iOR-ligand complexes: (a) *Dm*Orco-VUAA1, (b) *Dm*OR59b-DEET, (c) *Dm*OR85b-2-heptanone, (d) *Dm*OR22a-methyl octanoate, (e) *It*OR46-(*S*)-(-)-ipsenol and (f) *Of*OR3- (E)-11-tetradecenyl acetate.

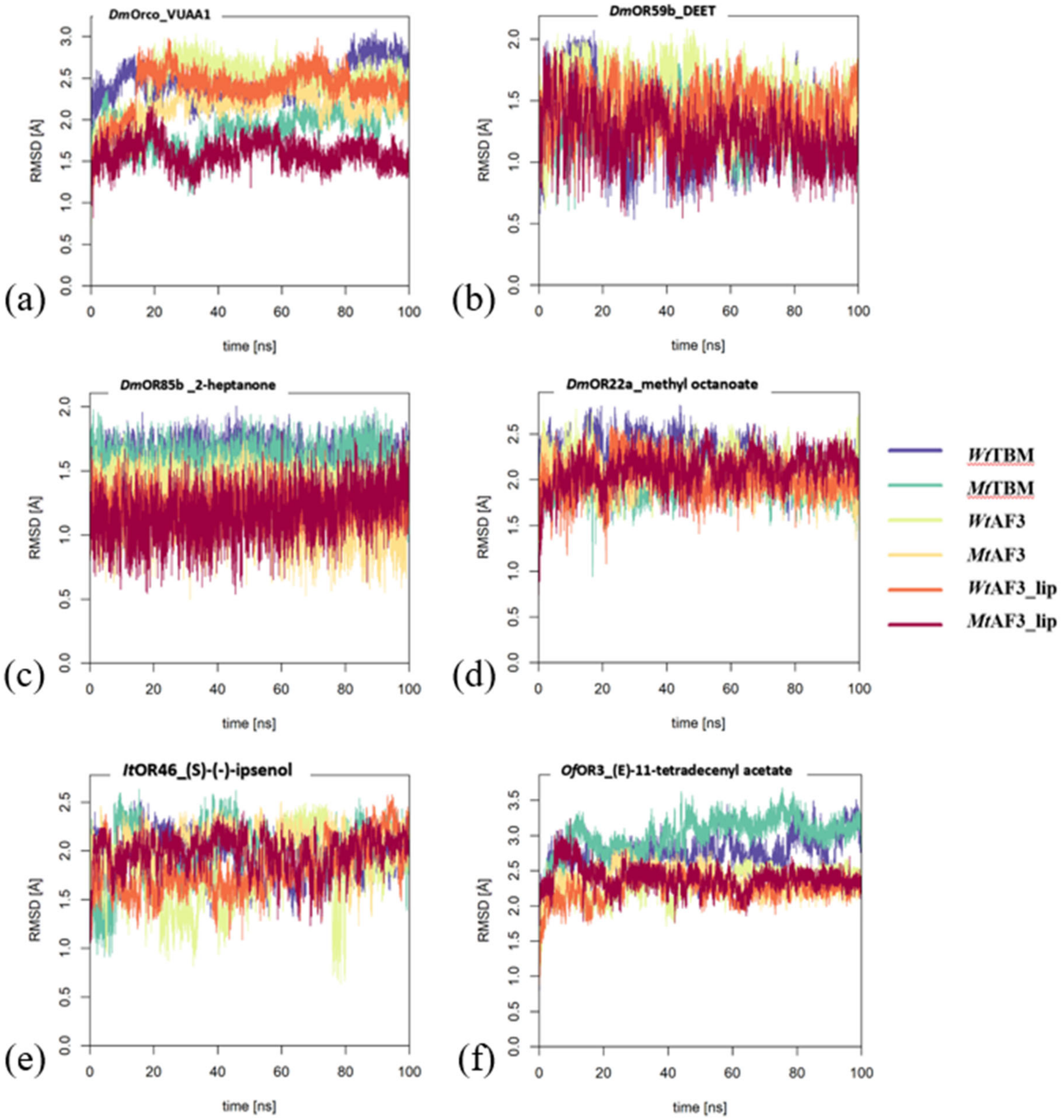

Figure S26. Ligand RMSDs of all the 36 predicted complexes over the last 100 ns. Average RMSD values of three replicates shown for (a) VUAA1 in the *Dm*Orco complex, (b) DEET in the *Dm*OR59b complex, (c) 2-heptanone in the *Dm*OR85b complex and (d) methyl octanoate in the *Dm*OR22a complex, (e) (*S*)-(-)-ipsenol in the *It*OR46 complex and (f) (E)-11-tetradecenyl acetate in *Of*OR3 complex.

Table S1: Experimental structures available for insect odorant receptors (iORs).

OR name	Species	PDBID	Ligand	Reference
*Ab*Orco	*Apocrypta bakeri*	6C70	-	[8]
*Mh*OR5	*Machilis hrabei*	7LIC	-	[9]
*Mh*OR5	*Machilis hrabei*	7LID	Eugenol	[9]
*Mh*OR5	*Machilis hrabei*	7LIG	DEET	[9]
ApOR5	*Acyrthosiphon pisum*	8Z9Z	-	[10]
ApOR5	*Acyrthosiphon pisum*	8Z9A	Geranyl acetate	[10]
AaOR10	*Aedes aegypti*	8V00	-	[11]
AaOR10	*Aedes aegypti*	8V02	*o*-cresol	[11]
AgOR28	*Anopheles gambiae*	8V3C	-	[11]
AgOR28	*Anopheles gambiae*	8V3D	2,4,5-trimethylthiazole	[11]

Table S2. iOR sequences and their respective selected templates used in this study.

No.	Sequence Name	Uniprot ID	Sequence length	Template selected with PDB ID and reference	Sequence identity (%) with the template
1	*Dm*Orco	Q9VNB5	486	*Ab*Orco (PDB ID: 6C70)	55.2
2	*Dm*OR59b	Q9W1P8	398	*Mh*OR5 (PDB ID: 7LIG)	12.9
3	*Dm*OR85b	Q9VHQ7	390	*Ap*OR5 (PDB ID: 8Z9A)	10.3
4	*Dm*OR22a	P81909	397	*Ap*OR5 (PDB ID: 8Z9A)	10.8
5	*It*OR46	A0A7L8XZ66	408	*Ap*OR5 (PDB ID: 8Z9A)	9.1
6	*Of*OR3	J7FCG8	422	*Mh*OR5 (PDB ID: 7LIG)	9.8

Table S3. Docking scores of all the 36 predicted models in this study.

**Docked receptor with ligand**	**Model approach**	**Docking scores (kcal/mol)**	
		**Wild**	**Mutant**
*Dm*Orco with VUAA1	TBM	**-7.5**	**-5.8**
	AF3	-7.1	-7.8
	AF3_lip	-7.6	-9.1
*Dm*OR59b with DEET	TBM	**-6.1**	**-1.7**
	AF3	**-5.8**	**-5.4**
	AF3_lip	-4.7	-6.2
*Dm*OR85b with 2-heptanone	TBM	**-5.4**	**-4.8**
	AF3	**-5.6**	**-5.2**
	AF3_lip	-5.6	-5.8
*Dm*OR22a with methyl octanoate	TBM	**-5.3**	**-4.8**
	AF3	**-5.1**	**-4.8**
	AF3_lip	-5.2	-5.5
*It*OR46 with (S)-(−)-ipsenol	TBM	**-6.4**	**-5.5**
	AF3	**-6.4**	**-5.8**
	AF3_lip	-5.7	-6.0
*Of*OR3 with (E)-11-tetradecenyl acetate	TBM	**-5.3**	**-6.1**
	AF3	-6.2	-5.4
	AF3_lip	**-6.4**	**-6.6**

Table S4. System size of all the 36 predicted complexes in this study.

**Complex**	**Modelling approach**	**System size (Å³)**	
		Wild	Mutant
*Dm*Orco_VUAA1	TBM	1009572 [34]	1006681 [34]
	AF3	914235	918476
	AF3_lip	1637616	1560521
DmOR59b_DEET	TBM	648547 [34]	682962 [34]
	AF3	691033	692087
	AF3_lip	693587	689978
*Dm*OR85b_2-heptanone	TBM	711960	711748
	AF3	742975	731665
	AF3_lip	743399	742378
*Dm*OR22a_methyl octanoate	TBM	738298	740284
	AF3	700209	700092
	AF3_lip	701641	702315
*It*OR46_(*S*)-(−)-ipsenol	TBM	657075	708650
	AF3	733245	739073
	AF3_lip	738446	728651
*Of*OR3_(E)-11-tetradecenyl acetate	TBM	674775	689958
	AF3	771995	770486
	AF3_lip	791347	790786

Table S5. MM/PBSA values (PB) and Standard Deviation (SD) of last 10 ns of the complexes (CSD) and their respective ligands (LSD) in all 36 complexes in this study. Replicates are labelled M1 - M5. Complexes marked with an asterisk (*) and in red font were not considered for analysis.

**Complexes**	**PB (kcal/mol)**	**CSD (last 10 ns)**	**LSD (last 10 ns)**	**PB (Mean)**	**SD of PB**	**CSD (Mean)**	**LSD (Mean)**
TBM_wt_*Dm*Orco_VUAA1_M1	-4.89	0.71	0.1	-6.51	1.49	0.42	0.18
TBM_wt_*Dm*Orco_VUAA1_M2	-7.83	0.29	0.25				
TBM_wt_*Dm*Orco_VUAA1_M3	-6.81	0.26	0.18				
TBM_mt_*Dm*Orco_VUAA1_M1	1.86	0.47	0.12	1.40	0.52	0.47	0.28
TBM_mt_*Dm*Orco_VUAA1_M2	1.50	0.52	0.48				
TBM_mt_*Dm*Orco_VUAA1_M3	0.83	0.42	0.23				
AF3_wt_*Dm*Orco_VUAA1_M2	1.60	0.26	0.18	1.09	0.47	0.30	0.17
AF3_wt_*Dm*Orco_VUAA1_M3	0.66	0.51	0.19				
AF3_wt_*Dm*Orco_VUAA1_M4	1.02	0.14	0.14				
AF3_wt_DmOrco_VUAA1_M1*	8.00	0.52	0.18				
AF3_mt_*Dm*Orco_VUAA1_M1	-7.70	0.20	0.10	-6.36	1.21	0.21	0.14
AF3_mt_*Dm*Orco_VUAA1_M2	-6.02	0.14	0.13				
AF3_mt_*Dm*Orco_VUAA1_M4	-5.35	0.29	0.19				
AF3_mt_*Dm*Orco_VUAA1_M3*	-4.09	0.18	0.07				
AF3-lip_wt_*Dm*Orco_VUAA1_M2	-5.91	0.18	0.14	-7.64	0.19	0.19	0.14
AF3-lip_wt_*Dm*Orco_VUAA1_M4	-7.20	0.17	0.20				
AF3-lip_wt_*Dm*Orco_VUAA1_M5	-9.80	0.21	0.09				

**Complexes**	**PB (kcal/mol)**	**CSD (last 10 ns)**	**LSD (last 10 ns)**	**PB (Mean)**	**SD of PB**	**CSD (Mean)**	**LSD (Mean)**
AF3-lip_wt_*Dm*Orco_VUAA1_M1*	-2.70	0.10	0.13				
AF3-lip_wt_*Dm*Orco_VUAA1_M3*	-1.01	0.2	0.13				
AF3-lip_mt_*Dm*Orco_VUAA1_M1	-5.5	0.11	0.24	-5.07	0.4	0.14	0.17
AF3-lip_mt_*Dm*Orco_VUAA1_M3	-4.7	0.13	0.15				
AF3-lip_mt_*Dm*Orco_VUAA1_M5	-5.01	0.17	0.12				
AF3-lip_mt_*Dm*Orco_VUAA1_M2*	-1.04	0.12	0.2				
AF3-lip_mt_*Dm*Orco_VUAA1_M4*	2.61	0.13	0.14				
TBM_wt_*Dm*OR59b_DEET_M1	-4.45	0.11	0.35	-4.8	0.85	0.15	0.29
TBM_wt_*Dm*OR59b_DEET_M2	-5.77	0.13	0.33				
TBM_wt_*Dm*OR59b_DEET_M3	-4.18	0.2	0.19				
TBM_mt_*Dm*OR59b_DEET_M1	3.15	0.29	0.23	3.31	0.49	0.39	0.2
TBM_mt_*Dm*OR59b_DEET_M2	3.86	0.68	0.26				
TBM_mt_*Dm*OR59b_DEET_M3	2.92	0.19	0.11				
AF3_wt_*Dm*OR59b_DEET_M1	2.13	0.18	0.17	1.46	0.72	0.11	0.2
AF3_wt_*Dm*OR59b_DEET_M3	1.54	0.08	0.29				
AF3_wt_*Dm*OR59b_DEET_M4	0.7	0.08	0.14				
AF3_wt_*Dm*OR59b_DEET_M2*	4.69	0.08	0.18				
AF3_mt_*Dm*OR59b_DEET_M2	1.26	0.13	0.21	2.7	1.87	0.13	0.16
AF3_mt_*Dm*OR59b_DEET_M3	2.04	0.15	0.19				
AF3_mt_*Dm*OR59b_DEET_M4	4.81	0.12	0.09				

**Complexes**	**PB (kcal/mol)**	**CSD (last 10 ns)**	**LSD (last 10 ns)**	**PB (Mean)**	**SD of PB**	**CSD (Mean)**	**LSD (Mean)**
AF3_mt_*Dm*OR59b_DEET_M1*	10.30	0.09	0.21				
AF3-lip_wt_*Dm*OR59b_DEET_M1	-4.44	0.11	0.39	-5.03	1.11	0.14	0.25
AF3-lip_wt_*Dm*OR59b_DEET_M3	-4.35	0.14	0.18				
AF3-lip_wt_*Dm*OR59b_DEET_M4	-6.31	0.16	0.18				
AF3-lip_wt_*Dm*OR59b_DEET_M2*	-0.21	0.12	0.22				
AF3-lip_mt_*Dm*OR59b_DEET_M1	-4.00	0.09	0.21	-6.10	1.82	0.11	0.24
AF3-lip_mt_*Dm*OR59b_DEET_M2	-7.10	0.09	0.23				
AF3-lip_mt_*Dm*OR59b_DEET_M4	-7.19	0.15	0.27				
AF3-lip_mt_*Dm*OR59b_DEET_M3*	-2.25	0.13	0.19				
TBM_wt_*Dm*OR85b_2-heptanone_M1	-9.00	0.08	0.14	-8.37	1.05	0.11	0.22
TBM_wt_*Dm*OR85b_2-heptanone_M2	-8.96	0.13	0.26				
TBM_wt_*Dm*OR85b_2-heptanone_M3	-7.16	0.13	0.25				
TBM_mt_*Dm*OR85b_2-heptanone_M1	-2.94	0.10	0.25	-3.08	1.62	0.11	0.27
TBM_mt_*Dm*OR85b_2-heptanone_M2	-4.76	0.12	0.3				
TBM_mt_*Dm*OR85b_2-heptanone_M3	-1.53	0.12	0.27				
AF3_wt_*Dm*OR85b_2-heptanone_M1	-8.00	0.12	0.20	-9.97	1.72	0.11	0.15
AF3_wt_*Dm*OR85b_2-heptanone_M2	-10.80	0.11	0.12				
AF3_wt_*Dm*OR85b_2-heptanone_M3	-11.12	0.1	0.12				

**Complexes**	**PB (kcal/mol)**	**CSD (last 10 ns)**	**LSD (last 10 ns)**	**PB (Mean)**	**SD of PB**	**CSD (Mean)**	**LSD (Mean)**
AF3_mt_*Dm*OR85b_2-heptanone_M2	-11.21	0.11	0.30	-10.84	0.77	0.13	0.30
AF3_mt_*Dm*OR85b_2-heptanone_M4	-9.96	0.10	0.29				
AF3_mt_*Dm*OR85b_2-heptanone_M5	-11.35	0.17	0.30				
AF3_mt_*Dm*OR85b_2-heptanone_M3*	-7.45	0.08	0.17				
AF3_mt_*Dm*OR85b_2-heptanone_M1*	-2.00	0.10	0.36				
AF3-lip_wt_*Dm*OR85b_2-heptanone_M1	-11.17	0.11	0.20	-12.76	1.64	0.10	0.14
AF3-lip_wt_*Dm*OR85b_2-heptanone_M2	-14.45	0.09	0.10				
AF3-lip_wt_*Dm*OR85b_2-heptanone_M3	-12.66	0.10	0.13				
AF3-lip_mt_*Dm*OR85b_2-heptanone_M1	-7.98	0.11	0.31	-7.28	1.14	0.10	0.26
AF3-lip_mt_*Dm*OR85b_2-heptanone_M3	-5.96	0.10	0.26				
AF3-lip_mt_*Dm*OR85b_2-heptanone_M4	-7.89	0.09	0.21				
AF3-lip_mt_*Dm*OR85b_2-heptanone_M2*	-3.88	0.09	0.27				
TBM_wt_*Dm*OR22a_methyl_octanoate_M1	-6.38	0.17	0.14	-5.84	1.60	0.14	0.17
TBM_wt_*Dm*OR22a_methyl_octanoate_M3	-7.10	0.14	0.16				
TBM_wt_*Dm*OR22a_methyl_octanoate_M5	-4.04	0.10	0.22				
TBM_wt_*Dm*OR22a_methyl_octanoate_M4*	-2.13	0.15	0.19				
TBM_wt_*Dm*OR22a_methyl_octanoate_M2*	-3.03	0.17	0.29				
TBM_mt_*Dm*OR22a_methyl_octanoate_M2	2.97	0.11	0.15	2.99	0.22	0.13	0.20
TBM_mt_*Dm*OR22a_methyl_octanoate_M3	2.78	0.09	0.18				
TBM_mt_*Dm*OR22a_methyl_octanoate_M4	3.22	0.19	0.28				
TBM_mt_*Dm*OR22a_methyl_octanoate_M1*	-1.90	0.09	0.14				

**Complexes**	**PB (kcal/mol)**	**CSD (last 10 ns)**	**LSD (last 10 ns)**	**PB (Mean)**	**SD of PB**	**CSD (Mean)**	**LSD (Mean)**
AF3_wt_*Dm*OR22a_methyl_octanoate_M3	-4.30	0.10	0.21	-3.6	0.96	0.10	0.24
AF3_wt_*Dm*OR22a_methyl_octanoate_M4	-3.99	0.10	0.22				
AF3_wt_*Dm*OR22a_methyl_octanoate_M5	-2.51	0.09	0.29				
AF3_wt_*Dm*OR22a_methyl_octanoate_M2*	-1.04	0.14	0.26				
AF3_wt_*Dm*OR22a_methyl_octanoate_M1*	-10.6	0.12	0.17				
AF3_mt_*Dm*OR22a_methyl_octanoate_M1	-6.09	0.08	0.16	-5.20	1.19	0.12	0.21
AF3_mt_*Dm*OR22a_methyl_octanoate_M2	-3.84	0.18	0.20				
AF3_mt_*Dm*OR22a_methyl_octanoate_M3	-5.66	0.11	0.27				
AF3-lip_wt_*Dm*OR22a_methyl_octanoate_M2	-4.59	0.12	0.19	-3.32	1.10	0.10	0.21
AF3-lip_wt_*Dm*OR22a_methyl_octanoate_M3	-2.75	0.09	0.22				
AF3-lip_wt_*Dm*OR22a_methyl_octanoate_M4	-2.63	0.09	0.22				
AF3-lip_wt_*Dm*OR22a_methyl_octanoate_M1*	3.84	0.13	0.23				
AF3-lip_mt_*Dm*OR22a_methyl_octanoate_M1	-8.00	0.13	0.17	-6.94	1.12	0.13	0.22
AF3-lip_mt_*Dm*OR22a_methyl_octanoate_M4	-5.76	0.13	0.20				
AF3-lip_mt_*Dm*OR22a_methyl_octanoate_M3	-7.06	0.13	0.30				
AF3-lip_mt_*Dm*OR22a_methyl_octanoate_M2*	-4.12	0.10	0.18				
TBM_wt_ItOR46_(S)-(-)-ipsenol_M1	-8.30	0.12	0.12	-7.30	1.48	0.13	0.23
TBM_wt_ItOR46_(S)-(-)-ipsenol_M2	-5.60	0.17	0.22				
TBM_wt_ItOR46_(S)-(-)-ipsenol_M3	-7.99	0.10	0.36				

**Complexes**	**PB (kcal/mol)**	**CSD (last 10 ns)**	**LSD (last 10 ns)**	**PB (Mean)**	**SD of PB**	**CSD (Mean)**	**LSD (Mean)**
TBM_mt_*It*OR46_(S)-(-)-ipsenol_M3	-0.20	0.15	0.31	-1.30	1.21	0.17	0.22
TBM_mt_*It*OR46_(S)-(-)-ipsenol_M4	-1.10	0.23	0.15				
TBM_mt_*It*OR46_(S)-(-)-ipsenol_M5	-2.60	0.14	0.21				
TBM_mt_*It*OR46_(S)-(-)-ipsenol_M1*	0.50	0.30	0.39				
TBM_mt_*It*OR46_(S)-(-)-ipsenol_M2*	1.89	0.23	0.15				
AF3_wt_*It*ypOR46_(S)-(-)-ipsenol_M1	-5.30	0.18	0.13	-5.34	0.25	0.16	0.23
AF3_wt_*It*ypOR46_(S)-(-)-ipsenol_M2	-5.60	0.15	0.41				
AF3_wt_*It*ypOR46_(S)-(-)-ipsenol_M3	-5.11	0.15	0.14				
AF3_mt_*It*OR46_(S)-(-)-ipsenol_M1	-2.61	0.09	0.32	-4.04	1.24	0.12	0.26
AF3_mt_*It*OR46_(S)-(-)-ipsenol_M2	-4.80	0.14	0.19				
AF3_mt_*It*OR46_(S)-(-)-ipsenol_M4	-4.71	0.14	0.27				
AF3_mt_*It*OR46_(S)-(-)-ipsenol_M3*	0.66	0.19	0.56				
AF3_lip_wt_*It*OR46_(S)-(-)-ipsenol_M1	1.46	0.12	0.24	2.19	0.70	0.22	0.31
AF3_lip_wt_*It*OR46_(S)-(-)-ipsenol_M2	2.27	0.24	0.38				
AF3_lip_wt_*It*OR46_(S)-(-)-ipsenol_M3	2.85	0.30	0.30				
AF3_lip_mt_*It*OR46_(S)-(-)-ipsenol_M1	-2.55	0.17	0.20	-3.15	1.39	0.24	0.19
AF3_lip_mt_*It*OR46_(S)-(-)-ipsenol_M2	-5.10	0.26	0.12				
AF3_lip_mt_*It*OR46_(S)-(-)-ipsenol_M3	-2.88	0.28	0.25				

**Complexes**	**PB (kcal/mol)**	**CSD (last 10 ns)**	**LSD (last 10 ns)**	**PB (Mean)**	**SD of PB**	**CSD (Mean)**	**LSD (Mean)**
TBM_wt_*Of*OR3_(E)-11-tetradecenyl acetate_M1	-6.70	0.14	0.39	-5.98	0.75	0.13	0.38
TBM_wt_*Of*OR3_(E)-11-tetradecenyl acetate_M2	-5.20	0.15	0.37				
TBM_wt_*Of*OR3_(E)-11-tetradecenyl acetate_M4	-6.05	0.11	0.37				
TBM_wt_*Of*OR3_(E)-11-tetradecenyl acetate_M3*	-1.87	0.19	0.16				
TBM_mt_*Of*OR3_(E)-11-tetradecenyl acetate_M1	-11.91	0.23	0.17	-11.56	0.57	0.25	0.17
TBM_mt_*Of*OR3_(E)-11-tetradecenyl acetate_M2	-11.88	0.31	0.20				
TBM_mt_*Of*OR3_(E)-11-tetradecenyl acetate_M3	-10.90	0.22	0.15				
AF3_wt_*Of*OR3_(E)-11-tetradecenyl acetate_M1	-19.55	0.08	0.16	-17.64	2.10	0.15	0.12
AF3_wt_*Of*OR3_(E)-11-tetradecenyl acetate_M2	-15.39	0.27	0.09				
AF3_wt_*Of*OR3_(E)-11-tetradecenyl acetate_M3	-17.98	0.11	0.11				
AF3_mt_*Of*OR3_(E)-11-tetradecenyl acetate_M1	-15.23	0.20	0.16	-16.83	1.96	0.29	0.14
AF3_mt_*Of*OR3_(E)-11-tetradecenyl acetate_M2	-19.02	0.45	0.12				
AF3_mt_*Of*OR3_(E)-11-tetradecenyl acetate_M3	-16.23	0.22	0.14				
AF3_lip_wt_*Of*OR3_(E)-11-tetradecenyl acetate_M1	-17.70	0.11	0.18	-17.76	1.82	0.09	0.16
AF3_lip_wt_*Of*OR3_(E)-11-tetradecenyl acetate_M4	-19.60	0.08	0.12				
AF3_lip_wt_*Of*OR3_(E)-11-tetradecenyl acetate_M3	-15.97	0.07	0.17				
AF3_lip_wt_*Of*OR3_(E)-11-tetradecenyl acetate_M2*	-5.48	0.12	0.15				
AF3_lip_mt_*Of*OR3_(E)-11-tetradecenyl acetate_M2	-14.96	0.17	0.16	-17.2	2.57	0.19	0.17
AF3_lip_mt_*Of*OR3_(E)-11-tetradecenyl acetate_M3	-16.63	0.23	0.11				
AF3_lip_mt_*Of*OR3_(E)-11-tetradecenyl acetate_M4	-20.00	0.18	0.24				

**Complexes**	**PB (kcal/mol)**	**CSD (last 10 ns)**	**LSD (last 10 ns)**	**PB (Mean)**	**SD of PB**	**CSD (Mean)**	**LSD (Mean)**
AF3_lip_mt_*Of*OR3_(E)-11-tetradecenyl acetate_M1*	-8.68	0.26	0.17				
AF3_lip_wt_*It*OR46_(S)-(-)-ipsenol_M1	1.46	0.12	0.24	2.19	0.70	0.22	0.31
AF3_lip_wt_*It*OR46_(S)-(-)-ipsenol_M2	2.27	0.24	0.38				
AF3_lip_wt_*It*OR46_(S)-(-)-ipsenol_M3	2.85	0.30	0.30				
AF3_lip_mt_*It*OR46_(S)-(-)-ipsenol_M1	-2.55	0.17	0.20	-3.15	1.39	0.24	0.19
AF3_lip_mt_*It*OR46_(S)-(-)-ipsenol_M2	-5.10	0.26	0.12				
AF3_lip_mt_*It*OR46_(S)-(-)-ipsenol_M3	-2.88	0.28	0.25				
